# CCR2 deficiency alters activation of microglia subsets in traumatic brain injury

**DOI:** 10.1016/j.celrep.2021.109727

**Published:** 2021-09-21

**Authors:** Kerri Somebang, Joshua Rudolph, Isabella Imhof, Luyi Li, Erene C. Niemi, Judy Shigenaga, Huy Tran, T. Michael Gill, Iris Lo, Brian A. Zabel, Gabriela Schmajuk, Brian T. Wipke, Stefka Gyoneva, Luke Jandreski, Michael Craft, Gina Benedetto, Edward D. Plowey, Israel Charo, James Campbell, Chun Jimmie Ye, S. Scott Panter, Mary C. Nakamura, Walter Eckalbar, Christine L. Hsieh

**Affiliations:** 1 Department of Medicine, Division of Rheumatology, University of California, San Francisco (UCSF), San Francisco, CA, USA; 2 San Francisco VA Health Care System, San Francisco, CA, USA; 3 School of Medicine, Lung Biology Center, Division of Pulmonology, UCSF, San Francisco, CA, USA; 4 Department of Medicine, Division of Endocrinology and Metabolism, UCSF, San Francisco, CA, USA; 5 Gladstone Institutes, San Francisco, CA, USA; 6 Palo Alto Veterans Institute for Research, Palo Alto, CA, USA; 7 Palo Alto VA Health Care System, Palo Alto, CA, USA; 8 Biogen, Cambridge, MA, USA; 9 ChemoCentryx, Mountain View, CA, USA; 10 Institute for Human Genetics, Department of Epidemiology and Biostatistics, Institute of Computational Health Sciences, University of California, San Francisco, San Francisco, CA, USA; 11 Parker Institute for Cancer Immunotherapy, San Francisco, CA, USA; 12 Chan Zuckerberg Biohub, San Francisco, CA, USA; 13 Department of Neurological Surgery, UCSF, San Francisco, CA, USA; 14 These authors contributed equally; 15 Senior author; 16 Present address: Moderna, Inc., Cambridge, MA, USA; 17 Lead contact

## Abstract

In traumatic brain injury (TBI), a diversity of brain resident and peripherally derived myeloid cells have the potential to worsen damage and/or to assist in healing. We define the heterogeneity of microglia and macrophage phenotypes during TBI in wild-type (WT) mice and *Ccr2*^−/−^ mice, which lack macrophage influx following TBI and are resistant to brain damage. We use unbiased single-cell RNA sequencing methods to uncover 25 microglia, monocyte/macrophage, and dendritic cell subsets in acute TBI and normal brains. We find alterations in transcriptional profiles of microglia subsets in *Ccr2*^−/−^ TBI mice compared to WT TBI mice indicating that infiltrating monocytes/macrophages influence microglia activation to promote a type I IFN response. Preclinical pharmacological blockade of hCCR2 after injury reduces expression of IFN-responsive gene, *Irf7*, and improves outcomes. These data extend our understanding of myeloid cell diversity and crosstalk in brain trauma and identify therapeutic targets in myeloid subsets.

## In brief

By single-cell RNA sequencing of traumatically injured and normal brains from wild-type and *Ccr2*^−/−^ mice, Somebang et al. define microglia, macrophage, and dendritic cell phenotypes in TBI. Targeting mouse and/or human CCR2 reduces specific TBI brain CNS myeloid compartments, dampens type I interferon responses, and improves cognition after TBI.

## Graphical Abstract

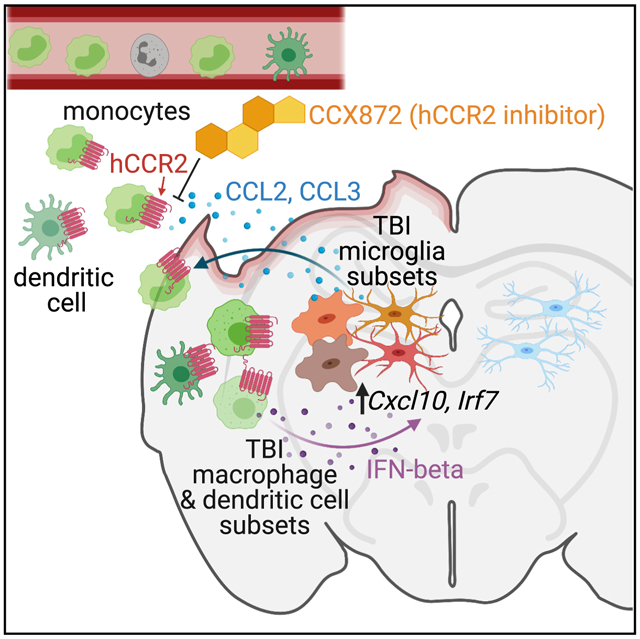

## INTRODUCTION

Traumatic brain injury (TBI) is a significant public health issue in the United States (US). In 2014, over 2.8 million TBI-related hospital visits and 50,000 deaths occurred in the US (Taylor et al., 2017). TBI often leads to lifelong motor, cognitive, and behavioral disabilities (Potts et al., 2006; Elder, 2015). It is also a risk factor for developing other neurodegenerative diseases, including Alzheimer’s and Parkinson’s disease (Elder, 2015).

In TBI, the mechanical insult to the brain rapidly elicits a series of potent immune responses in the central nervous system (CNS), collectively termed neuroinflammation (Potts et al., 2006; Corps et al., 2015; Hinson et al., 2015). The responding circulating immune cells include monocyte-derived macrophages, which infiltrate the brain and differentiate into activated macrophages within and around the area of injury. In addition, resident innate immune cells, including microglia, become activated at the injury site (Jassam et al., 2017). Although inflammation likely evolved to sterilize wounds and to facilitate repair (Liu et al., 2016; Russo et al., 2018), inflammation may remain active over years in humans and mice, resulting in secondary injury featuring neurotoxicity and expanding brain damage (Corps et al., 2015; Martin and Leibovich, 2005; Loane et al., 2014; Johnson et al., 2013; Ertürk et al., 2016). There is no effective pharmacological treatment for TBI, but the prolonged nature of secondary injury offers opportunities to intervene. We have sought to understand innate immune cells and pathways that are harmful or protective in TBI (Hsieh et al., 2013; Kim et al., 2016). Precisely identifying components of neuroinflammation, including immune cell subsets and activated pathways that impact TBI, will lead to informed designs for treatment.

Our TBI studies use an established model of controlled cortical impact (CCI) TBI in mice, which induces a focal brain injury and recapitulates key features of human TBI, including bleeding, blood-brain barrier (BBB) damage, edema, progressive loss of neurons, inflammatory leukocyte infiltration into the brain, cytokine upregulation, and functional deficits in animal behavior (Xiong et al., 2013; Hsieh et al., 2013, 2014; Jassam et al., 2017). We previously demonstrated that mice deficient in C-C chemokine receptor-2 (*Ccr2*) exhibit reduced macrophage infiltration, improved hippocampal-dependent cognitive outcomes, and preserved viable hippocampal neurons (Hsieh et al., 2014). Other studies similarly support an overall deleterious role for the recruitment of peripheral monocytes to the brain in TBI (Semple et al., 2010; Morganti et al., 2015). In humans, CCL2 protein is produced locally within hours post-TBI and remains elevated in the CSF for up to 9 days, demonstrating that this pathway is steadily active in acute human TBI (Semple et al., 2010).

While these studies demonstrated a role for CCR2 in the damaging effects of neuroinflammation following TBI, they left open the possibility that healing monocyte/macrophages are present but are overwhelmed by inflammatory myeloid cells. We therefore sought to define macrophage and microglial subtypes in TBI with the hypothesis that beneficial macrophages and/or phenotypes would emerge in the absence of CCR2. While a number of studies characterized macrophage and microglia responses following TBI in bulk cell population studies, the study of average expression obscures the actions of cellular subsets (Mahata et al., 2014; Patel et al., 2014; Shalek et al., 2014; Trapnell, 2015; Wu et al., 2014; Ricardo-Gonzalez et al., 2018). Recent studies have begun to unravel the heterogeneity of brain macrophage and microglia subsets primarily in chronic neurodegenerative diseases of Alzheimer’s disease and experimental autoimmune encephalitis, development, and aging (Jordão et al., 2019; Keren-Shaul et al., 2017; Hammond et al., 2019; Mathys et al., 2017; Sala Frigerio et al., 2019). To complement those studies, we identified myeloid cell subtypes in the setting of traumatic brain injury.

We report here the identification of microglia and macrophage subsets that arise following acute TBI. We identified alterations of microglia phenotypes that are associated with the functional benefit of targeting *Ccr2*, including a reduction in the type I IFN response. Because CCR2 is restricted to circulating immune cells, such as monocytes and dendritic cells, and it is not expressed by microglia (Mizutani et al., 2012), the effects of *Ccr2* deficiency on microglia are likely not direct and instead indicate the presence of crosstalk between microglia and infiltrating monocytes. Finally, we conducted translational studies to pharmacologically target human CCR2 with a small-molecule inhibitor in hCCR2 knockin mice. Blockade of hCCR2 2h after TBI blocked the infiltration of macrophages and partially blocked loss of cognitive function. This demonstrates the feasibility of treating TBI even after injury and shows that similar findings in mice genetically lacking *Ccr2* were not due to developmental influences. Our *in vivo* data corroborate our single-cell RNA sequencing (scRNA-seq) data to suggest that reducing the type I IFN response in microglia may also be a mechanism of neuroprotection.

## RESULTS

### Single-cell RNA sequencing identifies cell lineages and subtypes in the acute TBI brain

**Figure 1.**
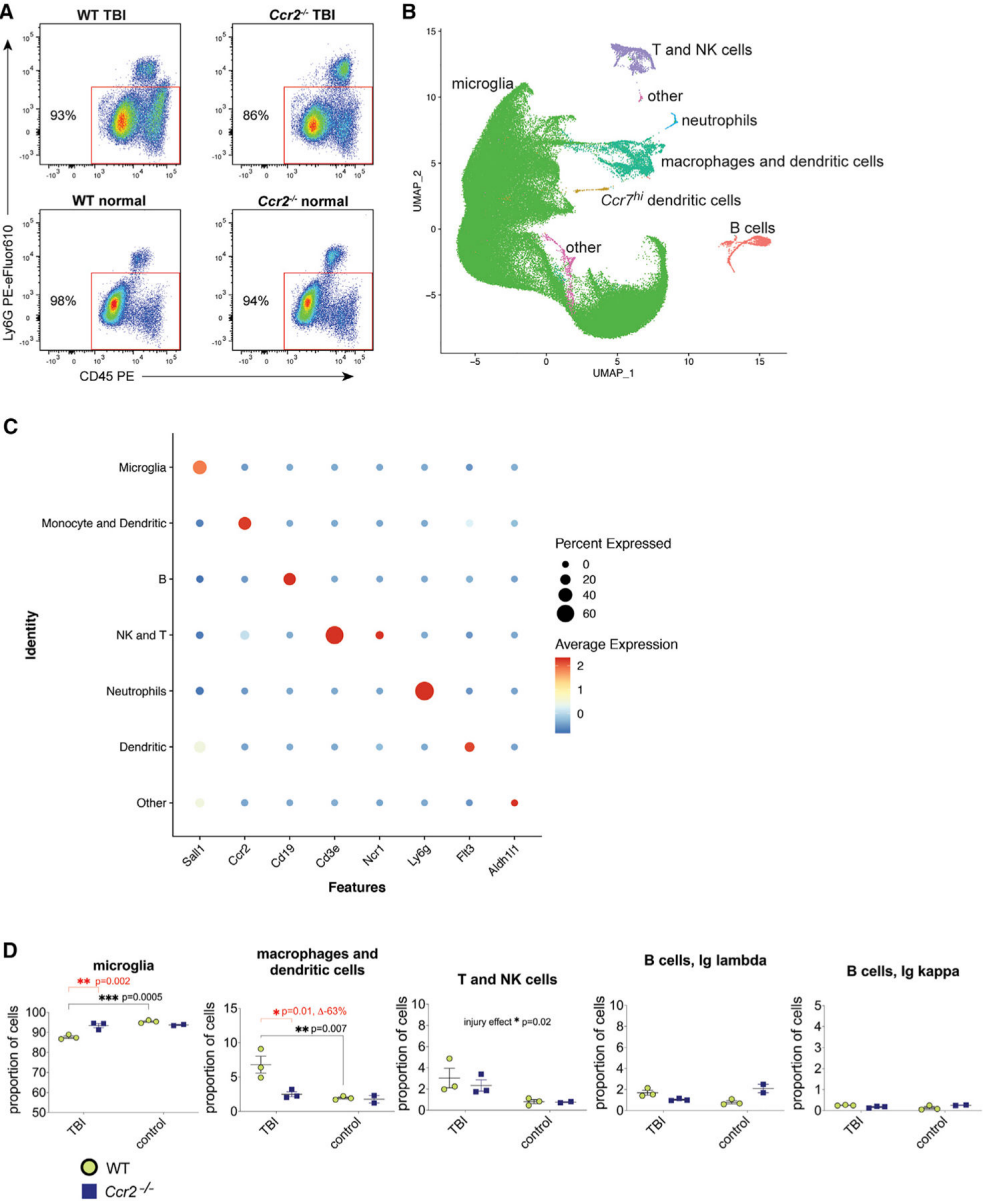
Defining cell lineages of clusters from scRNA-seq of white cells from ipsilateral acute TBI and normal brain tissues from *Ccr2*^−/−^ and WT mice (A) Representative flow-cytometry gates of LIVE CD45^+^Ly6G^−^ cells sorted from ipsilateral brain hemispheres from WT TBI mice (4 days post-injury, n = 3 animals, 1 animal/sample), *Ccr2*^−/−^ TBI mice (n = 3 animals, 1 animal/sample) and normal control mice (n = 2-3 animals/group, 1 animal/sample). (B) UMAP visualization of identified cell lineages of 111,717 cells from all animal groups combined. (C) Dot plot of cell-lineage marker expression that was used to help define clusters for microglia (*Sall1*), monocyte/macrophages (*Ccr2*), dendritic cells (*Flt3*), B cells (*Cd19*), T cells (*CD3e*), NK cells (*Ncr1*), neutrophils (*Ly6g*), neurons (*Slc12a5*), astrocytes (*Aldh1l1*), and oligodendrocytes (*Mog*). (D) Cell proportions of microglia and circulating leukocytes per brain hemisphere by genotype and by injury show that TBI induced increases in macrophages/dendritic cells. *Ccr2* deficiency robustly reduced the macrophage/dendritic cells in the ipsilateral brain tissue post-TBI.

We previously showed that *Ccr2*^−/−^ male mice demonstrated improvements in memory and histopathology following TBI, compared to wild-type (WT) mice (Hsieh et al., 2014). To better understand the mechanisms associated with this improvement, we sought to determine the differences in immune responses in acute male TBI between *Ccr2*^−/−^ mice and WT C57BL/6 mice. We analyzed brain samples 4 days post-TBI, a time point that we previously demonstrated to have peak infiltration of peripheral monocytes (Hsieh et al., 2014). We performed scRNA-seq on 111,717 high-quality cells combined from ipsilateral hemispheres of WT TBI mice (n = 3 animals, 1 animal/sample), *Ccr2*^−/−^ TBI mice (n = 3 animals, 1 animal/sample), and normal control mice (n = 2–3 animals/group, 1 animal/sample) from both genotypes. Sham controls can elicit low localized levels of inflammation and were used in our downstream validation experiments. We sorted cells by flow cytometry, gating for CD45^+^ Ly6G^−^ live singlets, which includes microglia and macrophages but excludes neutrophils (Figure 1A).

ScRNA-seq was performed, cell doublets were excluded, and data were analyzed from microglia and circulating leukocytes from 35,405 individual transcriptomes of WT cells after TBI, 28,918 *Ccr2*^−/−^ cells after TBI, 33,365 WT cells without TBI, and 14,029 *Ccr2*^−/−^ cells without TBI expressing an average of 22,701 genes/sample. Gene expression from WT and *Ccr2*^−/−^ cells were compared and aligned by using canonical correlation analysis (CCA) with Seurat (Butler et al., 2018). The high dimensional data were visualized by using UMAP (uniform manifold approximation and projection) dimensionality reduction technique (Becht et al., 2018) (Figure 1B). An analysis of cell-lineage markers to define cell clusters identified a large population of microglia (101,916 microglia), a cluster comprising a mix of monocytes/macrophages and dendritic cells, a separate *Ccr7*^*hi*^ dendritic cell subset (5,157 monocyte/macrophages/dendritic cells), lymphocyte clusters, and “other” unclear cell-lineage clusters (Figure 1A). Examples of cell-lineage markers used to define clusters include: microglia (*Sall1, Tmem119*) (Bennett et al., 2016; Buttgereit et al., 2016), monocyte/macrophages (*Ccr2, F13a1*) (Hammond et al., 2019), dendritic cells (*Flt3, Zbtb46*) (Merad et al., 2013), B cells (*Cd19*), T cells (*CD3e*), NK cells (*Ncr1*), neutrophils (*Ly6g*), neurons (*Slc12a5*), astrocytes (*Aldh1l1*), and oligodendrocytes (*Mog*) (Figures 1C and S1). In normal brains, a large majority of the cells identified were microglia as these samples lacked infiltrating leukocytes.

A gross analysis of cell proportions of all cells analyzed per half brain by genotype and by injury showed that TBI induced significant increases in the proportion of the macrophage/dendritic cells cluster (ANOVA p value = 0.007; WT TBI = 6.8% ± 2 [mean ± SD] versus WT normal control = 1.9 ± 0.2, adj p value = 0.007) (Figure 1D). Statistical analysis found a significant difference between WT and *Ccr2*^−/−^ cells in the microglia cluster and in the macrophage/dendritic cell cluster (ANOVAs p value = 0.01) (Figure 1D).

### Identification of microglia subsets in normal and acute TBI ipsilateral brains that are enriched in the type I IFN response, proliferation, disease-associated microglia markers, leukocyte migration pathways, and the IL-1 response

**Figure 2.**
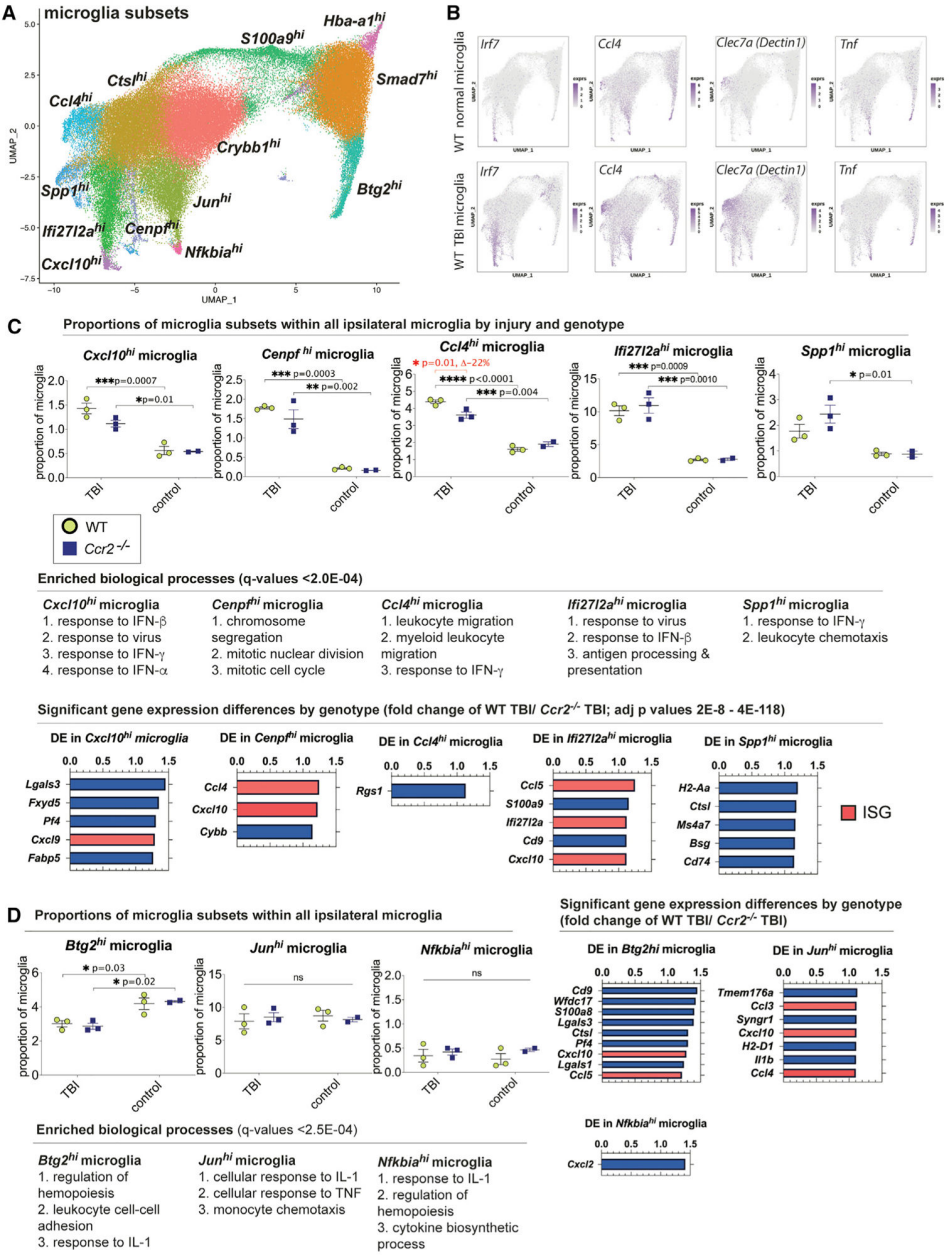
Quantification and gene-expression analysis of microglia subsets reveal subsets upregulated in TBI and pathways associated with *Ccr2* (A) ScRNA-seq and clustering analysis of all microglia (102,997 microglia analyzed) from all samples (n = 11 individual animals: n = 3 per TBI animal group; n = 2–3 per control animal group) separated the microglia into 13 distinct subsets as visualized by a UMAP. (B) Gene-expression plots of selected top DEGs. *Irf7* was a DEG of *Cxcl10*^*hi*^ and *Ifi27l2a*^*hi*^ microglia subsets, *Ccl4* and *Clec7a* were DEGS of *Ccl4*^*hi*^ microglia, *Tnf* was the top DEG for *Nfkbia*^*hi*^ microglia. *Irf7*-, *Ccl4*-, and *Clec7a*-expressing microglia were present in normal brain tissue and expanded in TBI. (C) Quantification of microglia subsets that significantly increased in proportion relative to all microglia analyzed during acute TBI compared to normal brains (n = 3/TBI group, n = 2–3/control group). The proportions of *Ccl4*^*hi*^ microglia were decreased in *Ccr2*^−/−^ TBI mice compared to WT TBI (p = 0.01). Proportions (top; mean ± SD) and pathway enrichment analysis (middle) for each microglia subset that increase in TBI are shown. Differential expression analysis between WT TBI and *Ccr2*^−/−^ TBI microglia subsets are shown (bottom). Many ISGs were consistently and significantly upregulated in WT TBI microglia subsets and are highlighted in salmon color (p = 2E-8 to 4E-118). (D) Analysis of a few microglia subsets that did not alter proportions relative to all microglia during TBI. Gene ontology (GO) analysis of DEGs of these subsets showed that they shared a response to IL-1. Some of these microglia showed transcriptional differences between WT and *Ccr2*^−/−^ mice. Differential expression analysis between WT TBI and *Ccr2*^−/−^ TBI microglia subsets revealed increased ISG expression in WT TBI *Btg2*^*hi*^ and *Jun*^*hi*^ microglia (right).

Joint graph-based clustering with canonical correlation vectors defined 13 distinct subclusters or subsets of microglia that were visualized by UMAP (Figure 2A). Each microglia subset was designated by a differentially expressed gene (DEG) with the highest fold change that distinguished that subset from all other microglia subclusters. The highest expressed DEGs possibly suggest some phenotype to its assigned subset, but we clarify that the DEGs were defined based on relative expression to other subclusters and were not necessarily exclusively expressed in its subset. The 13 microglia subsets were named as follows: (1) *Cxcl10*^*hi*^ (C-X-C motif chemokine ligand 10), (2) *Cenpf*^*hi*^ (centromere protein F), (3) *Ccl4*^*hi*^ (chemokine C-C motif ligands 4), (4) *Ifi27l2a*^*hi*^ (interferon alpha-inducible protein 27-like protein 2A), (5) *Spp1*^*hi*^ (secreted phosphoprotein 1), (6) *Btg2*^*hi*^ (B cell translocation gene 2), (7) *Jun*^*hi*^ (Jun proto-oncogene, AP-1 transcription factor subunit), (8) *Hba-a1*^*hi*^ (hemoglobin alpha, adult chain 1), (9) *Crybb1*^*hi*^ (crystallin beta B1), (10) *Smad7*^*hi*^ (SMAD family member 7), (11) *Nfkbia*^*hi*^ (NFKB inhibitor alpha), (12) *S100a9*^*hi*^ (S100 calcium binding protein A9), and (13) *Ctsl*^*hi*^ (cathepsin L) microglia. Gene expression of selected DEGs and microglia subclusters in normal mice were compared to those in mice following TBI. Shown are UMAPs of *Irf7*, which is a top 10 DEG of *Cxcl10*^*hi*^ and *Ifi27l2a*^*hi*^ microglia; *Ccl4* and *Clec7a* (Dectin 1), which are top 5 DEGs of *Ccl4*^*hi*^ microglia; and *Tnf*, which was the top DEG for *Nfkbia*^*hi*^ microglia (Figure 2B). The data demonstrated that each DEG highlighted a specific cluster and that the expression of *Irf7, Ccl4*, and *Clec7a* increased in TBI microglia compared to normal microglia, though there appears to be little to no increase in *Tnf*-expressing microglia (Figure 2B).

A heatmap showing the top 10 DEGs of each subset relative to other microglia demonstrated differences between the microglia subsets (Figure S2). We also performed gene ontology (GO) analysis of significant DEGs to begin to understand the phenotype of each microglia subset (Figures 2C, 2D, and S3A). There were five notable observations from the functional pathway analysis of enriched genes. The first is the presence of two microglia subsets, *Cxcl10*^*hi*^ and *Ifi27l2a*^*hi*^, with significantly enriched expression of genes from the type I interferon (IFN) pathway, including a 2- to 3-fold increase in the expression of a type I IFN-stimulated gene (ISG) and transcription factor, *Irf7* (interferon regulatory factor 7) (Ivashkiv and Donlin, 2014; Schneider et al., 2014) (Figures S2 and S3A). Although these two ISG-expressing microglia subsets share some genes, a direct comparison found clear distinctions between them (Figure S3B). The second observation is that the *Cenpf*^*hi*^ microglia were enriched in cell-cycle genes, including *Mki67* (marker of proliferation Ki67) (Figures S2 and S3A). A third important observation is that the *Ccl4*^*hi*^ microglia subset was enriched in several TREM2-dependent disease-associated microglia (DAM) marker genes (Keren-Shaul et al., 2017), including *Ccl6* (chemokine C-C motif ligand 6), *Lpl* (lipoprotein lipase), *Clec7a, Cd9, Cd63, Ctsb* (cathepsin B), *Ank* (progressive ankylosis), *Cst7* (cathepsin 7), and *Apoe* (apolipoprotein E) (Figures S2 and S3A). Fourth, multiple microglia subsets preferentially expressed genes with functions related to leukocyte migration, particularly for monocytes and neutrophils (Figures 2C, 2D, and S3A). And finally, three of the microglia subsets, *Btg2*^*hi*^, *Jun*^*hi*^, and *Nfkbia*^*hi*^, were enriched in IL-1 response genes (Figures 2D and S3A).

### TBI expands the proportions of microglia subsets that are responsive to type I IFNs and that express proliferation or DAM marker genes

We next determined the effects of TBI on the proportion and gene expression of each microglia subset relative to all microglia analyzed (Figure 2B; Figure 2C; Figure S4). Five of the 13 WT microglia subsets, *Cxcl10*^*hi*^, *Cenpf*^*hi*^, *Ccl4*^*hi*^, *Ifi27l2a*^*hi*^, and *Spp1*^*hi*^, were increased in proportion in WT TBI animals compared to normal controls (Figure 2B). Genes elevated following TBI were also evaluated for pathway enrichment analysis (Figure S5). Following TBI, proliferating *Cenpf*^*hi*^, *Ccl4*^*hi*^, *Spp1*^*hi*^, *Nfkbia*^*hi*^, and *Smad7*^*hi*^ microglia, expressed genes indicative of a response to IFN-β and/or IFN-α during TBI (Figure S5). TBI induced heterogeneous and distinct responses from microglia subsets.

### *Ccr2* deficiency alters TBI microglia subset proportions and transcription

To determine the effects of CCR2 deficiency on the proportion of each microglia subset, we compared WT TBI microglia to *Ccr2*^−/−^ TBI microglia from our scRNA-seq dataset. Interestingly, *Ccr2* deficiency reduced the proportion of *Ccl4*^*hi*^ microglia in *Ccr2*^−/−^ TBI mice by 22% compared to WT TBI brains (p = 0.01), while the other microglia were not significantly affected (Figure 2C). One microglia subset, *Hba-a1*^*hi*^ microglia, was increased in *Ccr2*^−/−^ TBI mice (Figure S4). The predicted functions for this subset by GO analysis, however, did not reveal any unique pathways (Figure S3A).

Differential expression analysis between WT TBI and *Ccr2*^−/−^ TBI microglia subsets revealed immune response pathways promoted by *Ccr2* in WT TBI mice. Compared to their corresponding *Ccr2*^−/−^ microglia subsets from TBI mice, four microglia subsets from WT TBI mice, *Cenpf*^*hi*^, *Ifi27l2a*^*hi*^, *Btg2*^*hi*^, and *Jun^hi^* microglia, preferentially expressed elevated levels of the ISG, *Cxcl10* with high statistical significance (adj p values = 3E-14, 4E-118, 5E-37, 2E-71, respectively) (Figures 2C, 2D, and S4). Other ISGs (Cheon et al., 2014), *Cxcl9, Ccl3, Ccl5*, and *Ifi27l2a*, were also significantly increased in WT TBI mice microglia subsets compared to *Ccr2*^−/−^ TBI microglia (adj p values = 0.01 to 2E-71) (Figures 2C and 2D). *Lgals3* (Galectin-3) was increased in *Cxcl10*^*hi*^, *Btg2*^*hi*^, *Crybb1*^*hi*^, *Smad7*^*hi*^, and *S100a9*^*hi*^ microglia subsets compared to *Ccr2*^−/−^ TBI microglia (adj p values = 3E-20 to 7E-111) (Figures 2C, 2D, and S4). Although the increase of gene expression in WT TBI mice was mild, ranging from 10%–45%, they were statistically highly significant and reproducible. In summary, *Ccr2* deficiency reduced the gene expression of ISGs and *Lgals3* in several microglia subsets.

### Type I IFN-responding microglia and a proliferating microglia subset localize to the TBI lesion site

**Figure 3.**
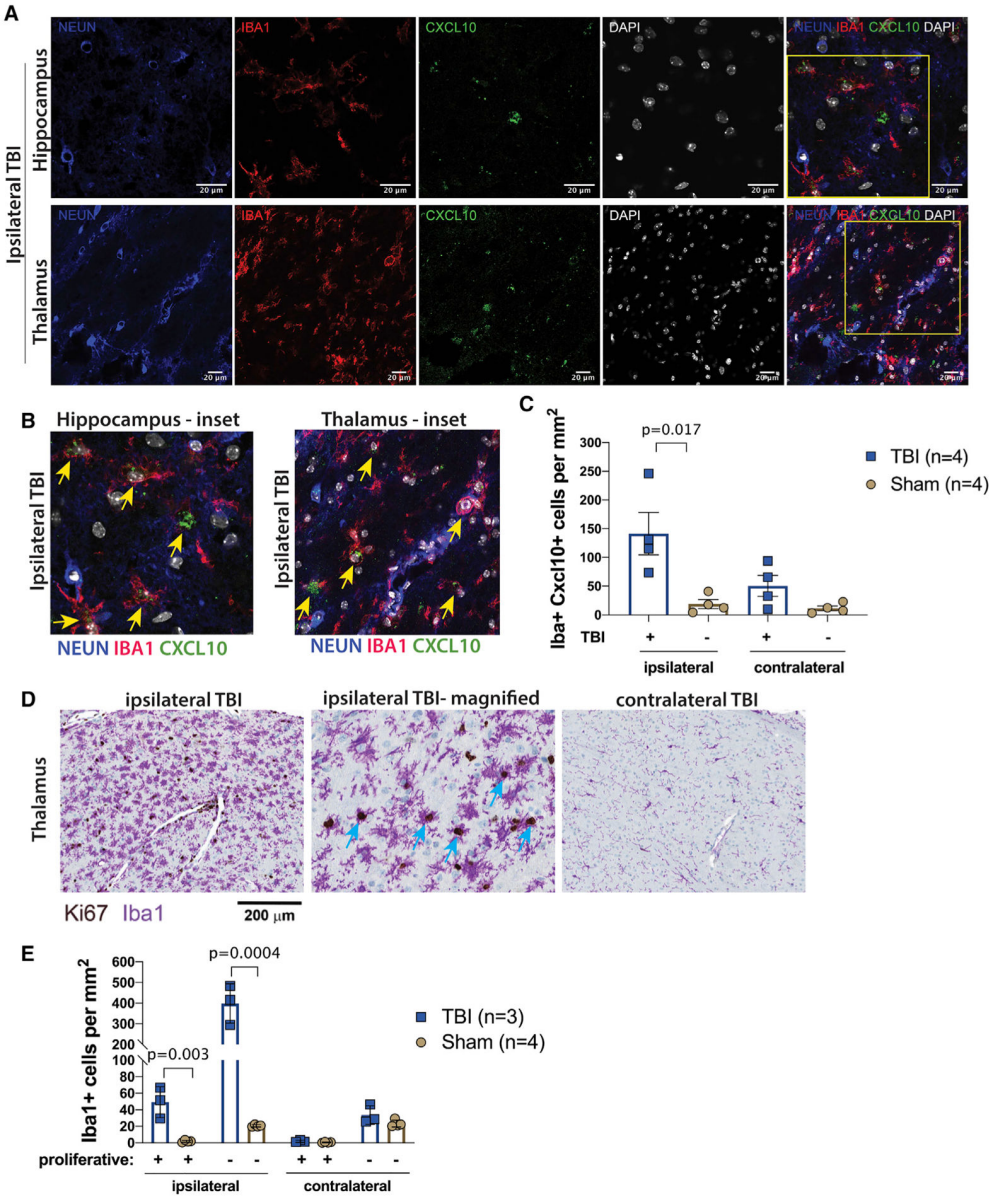
Histological validation and quantification of microglia subsets at the injury site (A) Four days after TBI or sham surgery, immunohistochemistry was performed on brain tissue sections with costaining for CXCL10 (green), IBA1 (red), NEUN (blue), and DAPI (white). The hippocampus and thalamic regions were analyzed. Scale bars indicate 20 μm. (B) CXCL10 and IBA1 co-localized in cells in the ipsilateral hippocampus and thalamus in magnified insets. (C) Quantification of IBA1^+^ CXCL10^+^ microglia/macrophages per square mm revealed a significant increase in cell numbers in the ipsilateral TBI hemisphere compared to sham animals (p = 0.017) (n = 4 TBI; n = 4 sham). (D) Immunohistochemistry on brain tissue post-TBI for the cell-proliferation marker, Ki67 (brown), and IBA-1 (purple). Scale bar indicates 200 μm for far-left and far-right images. (E) Quantification of Ki67 and IBA1 expressing cells demonstrated elevated proliferative (p = 0.003) and non-proliferative (p = 0.0004) microglia/macrophages in the ipsilateral TBI hemisphere compared to sham controls (n = 3 TBI; n = 4 sham).

To validate the identification and localization of at least two microglia subsets at the protein level, we performed immunohistochemistry. Histological analysis for expression of CXCL10, IBA1, NEUN, and DAPI showed co-expression of CXCL10 and IBA-1 in cells localized in the ipsilateral (side of injury) hippocampus and thalamus (Figures 3A and 3B). Quantification showed that ipsilateral TBI tissue had a significant increase in the mean number of IBA1^+^CXCL10^+^ cells compared to sham brain tissue (mean ± SD; 141 ± 73 versus 18 ± 15, p value = 0.017) (Figure 3C). No significant increase in CXCL10^+^ cells was observed between contralateral TBI and sham tissue (mean ± SD; 50 ± 36 versus 11 ± 8, p value = 0.08) (Figure 3C). These histology data support the findings in scRNA-seq that there is a CXCL10^+^ microglia subset that increases in cell number in the ipsilateral hemisphere of the injured CNS.

Immunohistochemistry for the cell-proliferation marker, Ki67, revealed robust expression of Ki67 in a subset of IBA1^+^ cells that resembled ramified, activated microglia localized in the thalamus of TBI animals (Figure 3D). Quantification demonstrated a significant increase of double-positive Ki67^+^IBA1^+^ cells in the ipsilateral thalamus, but not the contralateral or sham control thalami (mean ± SD; 49 ± 18 versus 2 ± 1, p value = 0.003) (Figure 3E). Thus, acute TBI induces the rise of CXCL10^+^ and Ki67^+^ subsets of IBA1-expressing cells near the site of brain injury in the thalamus. Histology results bolster the scRNA-seq data and demonstrate that there is a proliferating subset of microglia that increases in TBI. These data extend the scRNA-seq findings to pinpoint the thalamus as a location in the CNS where microglia subsets expand in CCI.

### Identification of monocyte/macrophage and dendritic cell subsets in normal and acute TBI ipsilateral brains revealed subsets enriched in pathways of response to type I IFN, of wound healing, and of leukocyte migration

**Figure 4.**
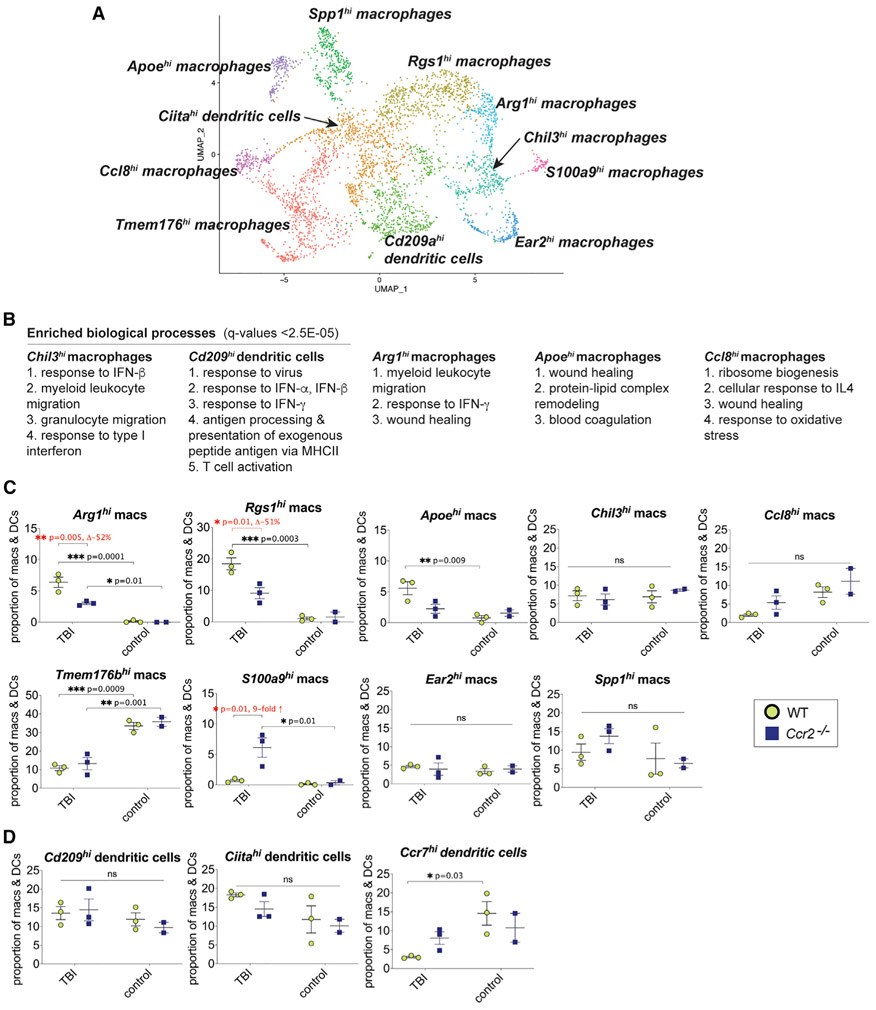
Monocyte/macrophage and dendritic cell subcluster analysis (A) UMAP visualization of nine monocyte/macrophage subclusters and two dendritic cell subclusters in addition to the *Ccr7*^*hi*^ dendritic cell cluster from all animal groups (n = 11 individual animal samples: n = 3 per TBI animal group; n = 2–3 per control animal group. The total number of monocyte/dendritc cells analyzed were 4,076). (B) GO analysis of five selected monocyte/macrophage and dendritic cell subclusters showed that macrophage/dendritic cells are enriched in type I IFN response genes and wound-healing genes. (C and D) Quantification of proportions of monocyte/macrophage subsets (C) and proportions (D) of dendritic cell subsets (mean ± SD) in TBI as a percentage of all monocyte/macrophages/dendritic cells analyzed.

The cell cluster expressing both monocyte/dendritic cell markers in Figure 1 was dissected further. Reclustering analysis with Seurat identified nine monocyte/macrophage clusters designated as *Rgs1*^*hi*^ (regulator of G protein signaling 1), *Arg1*^*hi*^ (arginase 1), *Chil3*^*hi*^ (chitinase-like 3), *Spp1*^*hi*^ (secreted phosphoprotein 1), *Tmem176b*^*hi*^ (transmembrane protein 176B), *Ear2*^*hi*^ (eosinophil-associated, ribonuclease A family, member2), *Apoe*^*hi*^ (apolipoprotein E), *Ccl8*^*hi*^ (C-C motif chemokine ligand 8), and *S100a9*^*hi*^ (S100 calcium binding protein A9) (Figure 4A). In addition to the *Ccr7*^*hi*^ dendritic cells identified in Figure 1, reclustering found two additional dendritic cell clusters designated as *Ciita*^*hi*^ (class II major histocompatibility complex transactivator), and *Cd209a*^*hi*^ (also known as DC-SIGN), both of which co-express the dendritic cell markers (Merad et al., 2013), *Flt3* (fms related receptor tyrosine kinase 3), *Itgax* (integrin subunit alpha X, CD11c), and *Zbtb46* (zinc finger and BTB domain containing 46) (Figure 4A). DEGs of each subset were visualized with a heatmap (Figure S6).

GO analysis found that *Ly6c2* was highly expressed in *Chil3*^*hi*^ monocytes (Figure S7), suggesting that these might be pro-inflammatory Ly6C^hi^ monocytes. We also found ISG expression in two subclusters, *Chil3*^*hi*^ monocytes and *Cd209a*^*hi*^ dendritic cells, suggesting the presence of active type I IFN pathways (Figures 4B and S7). Four of the monocyte/macrophage subsets, *Arg1*^*hi*^, *Rgs1*^*hi*^, *Apoe*^*hi*^, and *Ccl8*^*hi*^, were enriched in wound-healing processes (Figures 4B and S7). Interestingly, the *Apoe*^*hi*^ macrophage subset also expressed DEGs similar to DAM (Keren-Shaul et al., 2017) and lipid-associated macrophages (LAMs) (Jaitin et al., 2019) and were enriched in pathways for remodeling protein-lipid complexes (Figures 4B and S7). We observed that *Ccl8*^*hi*^ macrophages expressed little to no *Ccr2* but expressed relatively high levels of *Mrc1* and *Lyve1*, suggesting that they could represent brain resident CNS-associated macrophages (CAMs) (Jordão et al., 2019). Leukocyte chemotaxis pathways were found among *Chil3*^*hi*^, *Arg1*^*hi*^, *Rgs1*^*hi*^, and *S100a9hi* macrophages (Figures 4B and S7). All three dendritic cells clusters, *Ciita*^*hi*^, *Cd209a*^*hi*^, and *Ccr7*^*hi*^, were enriched in genes associated with T cell activation (Figure S7). CNS injured macrophages and dendritic cell phenotypes were also diverse.

### *Ccr2* deficiency culls specific monocyte/macrophage subsets normally elevated in the CNS by TBI

TBI resulted in the expansion cell proportions of three out of the nine identified monocyte/macrophage subsets, *Arg1*^*hi*^, *Rgs1*^*hi*^, and *Apoe*^*hi*^ macrophages (Figure 4C). Notably, *Arg1*^*hi*^*, Rgs1*^*hi*^ monocyte/macrophages were significantly attenuated in *Ccr2*^−/−^ TBI mice compared to WT TBI mice showing ~50% reduction in mean proportions (adj p values = 0.005 and 0.01, respectively) (Figure 4C). The individual role and impact of each subset are yet to be determined.

### Validation of Ly6C^hi^ and *Chil3* co-expression on TBI monocyte/macrophages

*Ly6c2* was identified as a DEG in *Chil3^hi^* macrophages with 2-fold higher expression compared to other subsets (Figure S7). This was unexpected, since high expression of Ly6C is a classic marker for proinflammatory monocytes (Shi and Pamer, 2011; Swirski and Nahrendorf, 2018), while *Chil3* is associated with anti-inflammation M(IL-4) (Murray et al., 2014; Chawla, 2010; Loke et al., 2002). Further, our data in Figure 4 revealed that *Chil3^hi^* and *Arg1^hi^* were markers of different subsets, which was another unexpected finding because *Chil3* and *Arg1* are signature markers that correlate in M(IL-4) macrophages (Murray et al., 2014; Chawla, 2010; Loke et al., 2002).

**Figure 5.**
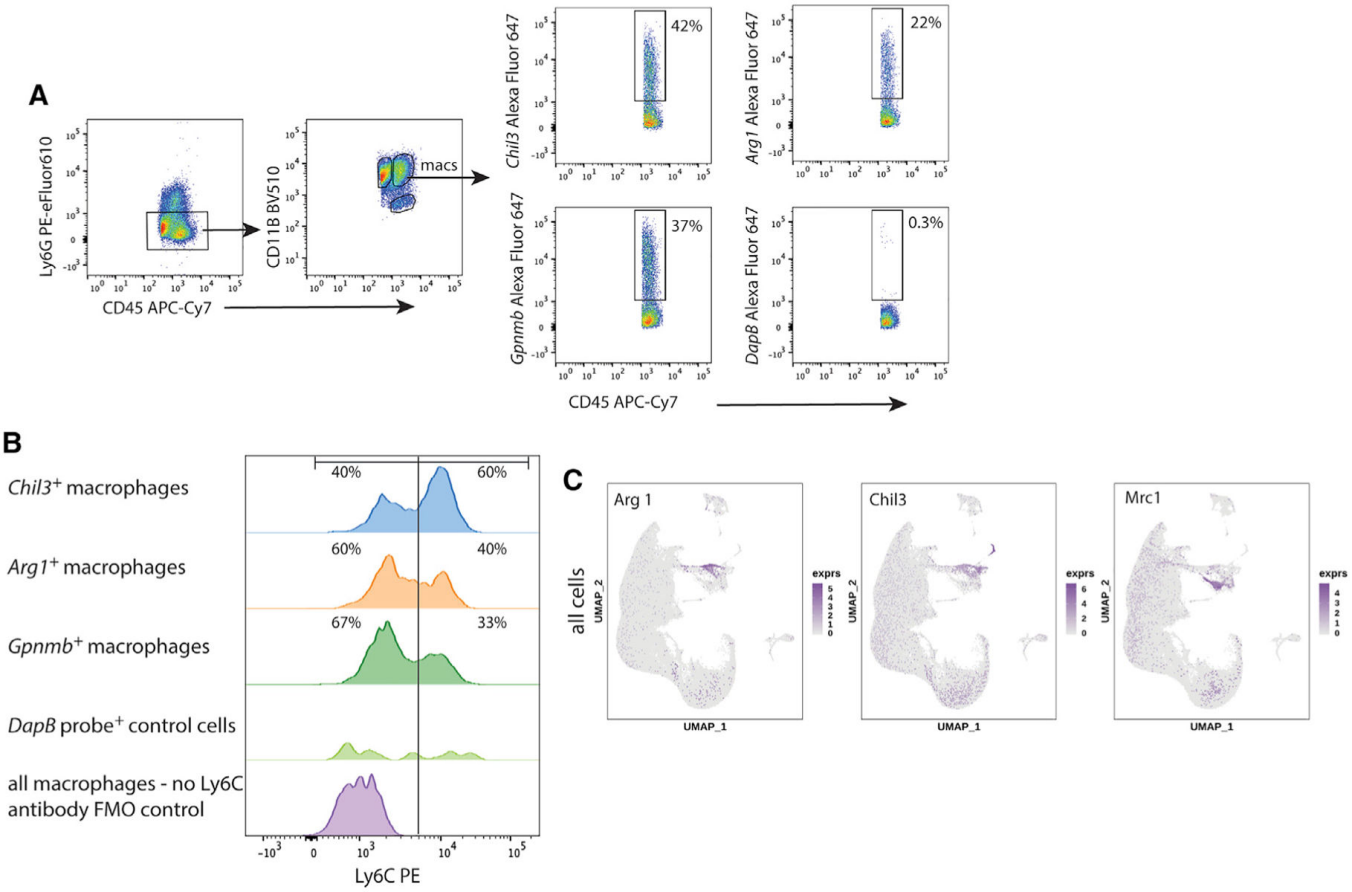
Validation of Ly6C^hi^ protein co-expression with *Chil3* in TBI macrophage subsets by flow cytometry (A) Flow-cytometry gating strategy for TBI day 4 ipsilateral brain white cells that are LIVE, CD45^hi^CD11b^+^, and Ly6G. Cells were further gated for their binding to RNA probes for *Chil3, Arg1, Gpnmb*, and a control RNA probe for *Dapb* (data are representative of three independent experiments). (B) Histogram analysis for Ly6C surface expression on gated TBI macrophages showed that Ly6C was preferentially highly expressed in 60% of *Chil3*^+^ TBI macrophages by flow cytometry. *Arg1* expression was distinct from *Chil3* expression as *Arg1* was predominantly found in Ly6C^lo^ cells. *Gpnmb* served as a co-expression marker for *Arg1*. FMO, fluorescence minus one. (C) UMAP visualization of all cells as in Figure 1B and their expression of signature M(IL-4) genes, *Arg1*, *Chil3*, and *Mrc1*.

To follow up on our datasets, we determined whether we could identify Ly6C^hi^ classic and Ly6C^lo^ nonclassic macrophages at the protein level in acute TBI in *Chil3^hi^* and *Arg1^hi^* macrophages (Figures 5A and 5B). Four days after TBI, brain leukocytes were isolated and flow cytometry was performed. We confirmed that Ly6C RNA and protein expression correlated and that Ly6C was highly expressed on 60% of *Chil*^+^ TBI macrophages (Figures 5A and 5B). Conversely, *Arg1* was predominantly found in Ly6C^lo^ monocyte/macrophages (Figure 5B). We also found that *Gpnmb* (glycoprotein nmb) served as a robust co-expression marker for *Arg1* (Figure 5B). The control RNA probe, *Dapb*, which detects a bacterial gene did not bind TBI macrophages (Figure 5A) and did not correlate with Ly6C (Figure 5B). While there is partial overlap between *Chil3* and *Arg1* expression (Figures 5B and 5C), they define distinct Ly6C expressing macrophage subsets (Figures 5B, 5C, and S7). Early scRNA-seq from our laboratory on *ex vivo* TBI macrophages demonstrated a lack of correlation between M1/M2 markers in TBI (Kim et al., 2016; Ransohoff, 2016). In our current expanded scRNA-seq dataset, we found that high expression of each signature M(IL-4) genes, *Arg1, Chil3*, and *Mrc1*, defined different subsets (Figure 5C). It is also notable that *Arg1, Chil3*, and *Mrc1* were not observed to correlate or serve as a DEG for any microglia subcluster (Figure 5C) supporting the insufficiencies of applying an M1/M2 paradigm to both mouse macrophages and microglia *in vivo*.

### Targeting hCCR2 pharmacologically after TBI reduces the type I IFN response *in vivo* and improves cognitive function

**Figure 6.**
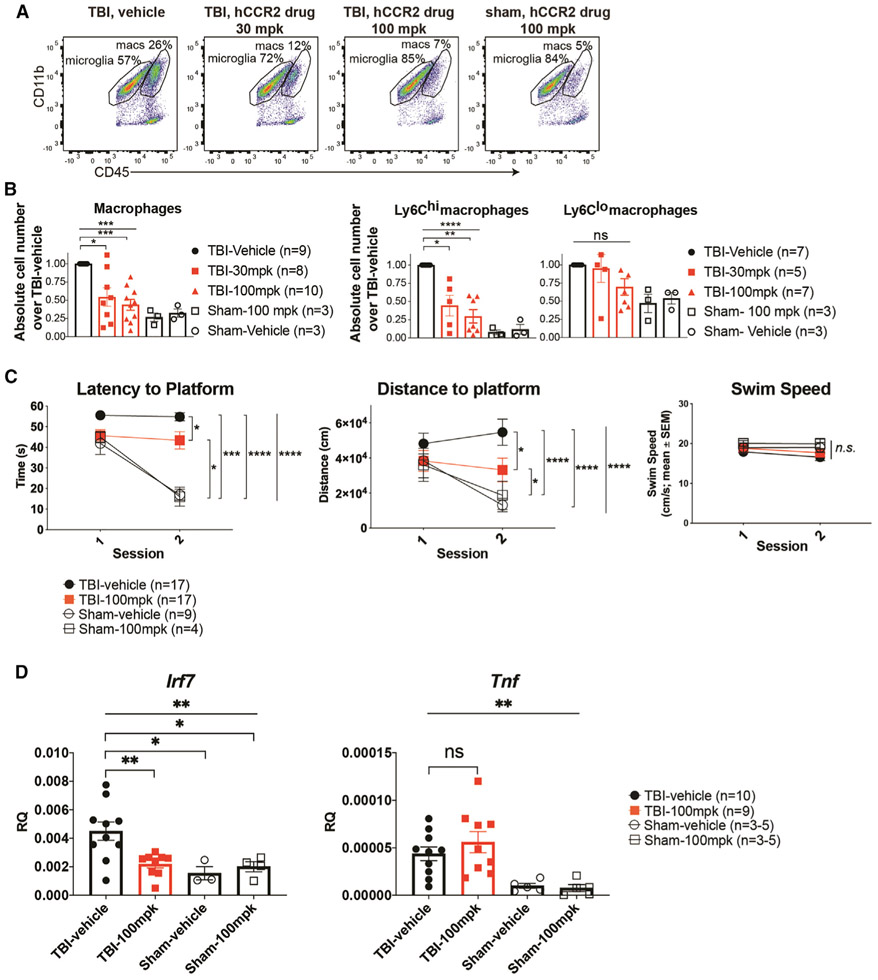
Targeting hCCR2 pharmacologically after TBI in hCCR2 knockin mice led to reduced macrophage infiltration into the brain, improved cognitive memory, and reduced expression of a key ISG, *Irf7* (A) hCCR2 knockin mice were administered CCX872 at 30 mg/kg (mpk), 100 mpk, or vehicle beginning 2 h post-surgery. Flow-cytometry analysis of microglia and macrophages proportions in the brain 1 day after surgery are shown. Ly6G^−^ viable cells are shown. Data represent at least three independent experiments. (B) Absolute macrophage numbers in ipsilateral hemispheres 4 days post-TBI or sham surgery were quantified by flow cytometry (n = 8–10 per TBI group; n = 3 per sham group) (*p < 0.05, **p < 0.01, ***p < 0.001, ****p < 0.0001). (C) A cued platform version of the Morris water maze was performed beginning 4 weeks post-surgery (n = 17 per TBI group; n = 4–9 per sham group). Data are shown with statistics reflecting rank summary score analysis. (D) Relative gene-expression analysis of ipsilateral brain hemispheres 4 days post-TBI or sham surgery was performed in triplicate with biological replicates (n = 9–10 per TBI group; n = 3–5 per sham group). Two independent experiments were performed.

Morganti et al. previously showed that a small-molecule inhibitor against human CCR2 (hCCR2) (Sullivan et al., 2013; Chen et al., 2016; Miao et al., 2018), CCX872, improved TBI outcomes in WT mice (Morganti et al., 2015) when it is administered prior to trauma. We examined the potential clinical use of this inhibitor in TBI by testing the hCCR2 inhibitor for efficacy when given 2 hours after trauma. Our studies used h*CCR2* knockin mice, in which human *CCR2* replaces the mouse *Ccr2* gene (Sullivan et al., 2013). Using human receptor transgenic mice was also important because the affinity of the drug is 100-fold greater for hCCR2 than its affinity for the mouse receptor. We quantified CD45^hi^CD11b^+^ macrophages infiltrating the brain 1 day post-TBI in hCCR2 knockin mice treated subcutaneously with drug or vehicle (n = 5–10 per TBI group, n = 3 per sham group). Representative flow-cytometry data of ipsilateral brain leukocytes showed that CCR2 blockade with 30 and 100 mg/kg (mpk) of CCX872 reduced macrophage infiltration by 46% ± 13% (mean ± SEM) and by 57% ± 8%, respectively (TBI vehicle versus TBI 30 mpk *p < 0.05; TBI vehicle versus TBI 100 mpk ***p < 0.001) (Figures 6A and 6B). CCX872 treatment significantly reduced the absolute cell numbers of Ly6C^hi^ macrophages in the ipsilateral brain by 56% ± 14% (30 mpk) and 71% ± 9% (100 mpk) (ANOVA ****p < 0.0001; TBI vehicle and TBI 30 mpk *p < 0.05; TBI vehicle versus TBI 100 mpk **p < 0.005) (Figure 6B). Additionally, we observed no differences at this early time point in absolute cell numbers of microglia, T cells, and neutrophils with drug treatment (Figure S8A).

To determine whether hCCR2 blockade after TBI improved cognitive outcomes, we next examined the effect of CCX872 treatment on behavior, as assessed by a cued platform version of Morris Water Maze testing at 4 weeks after injury (Hsieh et al., 2014; Patil et al., 2009; Vorhees and Williams, 2006; Possin et al., 2016) (Figure 6C). Mice were given TBI or sham surgery followed by administration of vehicle or hCCR2 inhibitor at 100 mpk beginning 2 h post-injury and then daily thereafter for 5 days (n = 17 per TBI group, n = 4–9 per sham group). Mice were assessed for their ability to learn the location of an escape platform with a visible cue on top. Mice treated with 100 mpk of the hCCR2 inhibitor performed better than mice treated with vehicle, both in escape latency (time to platform) and total distance from the platform (Figure 6C) (rank summary scores [Possin et al., 2016] over both sessions: ANOVA ****p < 0.0001 for both latency and distance; TBI vehicle versus TBI 100 mpk *p = 0.033 for latency and *p = 0.023 for distance). The swim velocities for all groups were equivalent, ruling out potential confounding factors related to swimming ability (Figure 6C). We also examined animal behavior using rotarod and open-field testing and found no significant differences between drug and vehicle-treated TBI animals (Figure S8B). Thus, acute treatment of mice with CCX872 was sufficient for significant recovery of cognitive function at delayed time points.

We next tested whether pharmacologic blockade of CCR2 reduced the type I IFN response by using quantitative RT-PCR on injured brain hemispheres 4 days after TBI (n = 9–10 per TBI group, n = 3–5 per sham group). TBI increased expression of a key ISG, *Irf7*, by an average 3-fold change (sham vehicle, mean relative quantification [RQ] to GAPDH = 4.5E^−3^; TBI vehicle, mean RQ = 1.5E^−3^; SEM = 1.2E^−3^; ANOVA **p < 0.001; post hoc test *p = 0.023). hCCR2 blockade reduced the increase in *Irf7* expression by 52% compared to the TBI vehicle-treated group (TBI vehicle RQ = 4.5E^−3^; TBI 100 mpk drug RQ = 2.1E^−3^; SEM = 7E^−4^; ANOVA **p < 0.001; **p = 0.005) (Figure 6D). In contrast, there was no difference observed in the expression of *Tnf*, which is not an ISG, between drug- and vehicle-treated animals (Figure 6D). The reduction in Ly6C^hi^ monocyte cell numbers in the TBI brain (Figure 6B) also indicates a reduction in *Chil3*^*hi*^ TBI macrophages, which have a pronounced type I IFN gene-expression profile (Figure 4B) and thus is likely contributing to the reduced IFN response in *Ccr2*^−/−^ TBI mice. These data support our findings from *Ccr2*^−/−^ mice that CCR2 intensifies the type I IFN response in TBI.

## DISCUSSION

Our scRNA-seq of cells from ipsilateral brain tissues post-acute TBI and normal brain tissues expands our understanding of myeloid cell diversity in the CNS and in TBI. We identify 25 distinct myeloid cell phenotypes. Importantly, we validate the protein expression of key microglia markers, CXCL10 and Ki67, in subsets of IBA1^+^ TBI microglia/macrophages in the ipsilateral hippocampus and thalamus by histology and quantify their expansion in the lesion area in TBI. This is consistent with previous findings of Cxcl10^+^ clusters being found in TBI (Israelsson et al., 2010). We validate heightened protein expression of Ly6C on *Chil3*^*hi*^ macrophages. By using ARG1 reporter mice, our group previously confirmed at the protein level that ARG1 clearly marked a subset of infiltrating F4/80^+^ monocyte/macrophages in TBI (Hsieh et al., 2013). Further validation of additional myeloid subsets with protein readouts and their spatial transcriptomic localization and defining their functional roles will be necessary to bring forth the potential vision of precisely modulating them for desired outcomes. Further analysis of sex differences across myeloid subsets will also be critical.

It is interesting that some myeloid subsets share similar phenotypes, such as the type I IFN response for *Cxcl10*^*hi*^ and *Ifi27l2a*^*hi*^ microglia. Multiple macrophage subsets are also enriched in wound healing processes, including *Arg1*^*hi*^, *Rgs1*^*hi*^, *Apoe*^*hi*^, and *Ccl8*^*hi*^ macrophages. Potential functional redundancies and/or distinctions between the subsets, and whether or not they represent transient transitional states, remain to be determined.

Importantly, we identify myeloid subsets that expand in TBI relative to their lineage; eight of them significantly increase in proportion in TBI: *Cxcl10*^*hi*^ microglia, *Cenpf*^*hi*^ microglia, *Ccl4*^*hi*^ microglia, *Ifi27l2a*^*hi*^ microglia, *Spp1*^*hi*^ microglia, *Arg1*^*hi*^ macrophages, *Rgs1*^*hi*^ macrophages, and *Apoe*^*hi*^ macrophages. Furthermore, TBI microglia subsets are similar based on marker expression to previously identified myeloid subsets, some with critically identified functions *in vivo*. *Ccl4*^*hi*^ TBI microglia share markers with disease-associated microglia (DAM); *Cenpf^hi^* TBI microglia are proliferating microglia; *Apoe*^*hi*^ TBI macrophages share markers with DAM and lipid-associated macrophages; *Ccl8*^*hi*^ TBI macrophages share defining markers with *Mrc1^+^ Lyve1^+^* resident CNS-associated macrophages, thus connecting the biology of TBI with findings in other neurodegenerative diseases (Jordão et al., 2019; Li et al., 2019; Jaitin et al., 2019; Hammond et al., 2019; Keren-Shaul et al., 2017), and acute injuries (Wahane et al., 2021). Mathys et al. and Frigerio et al. report type I IFN responsive and proliferating microglia in Alzheimer disease (Sala Frigerio et al., 2019; Mathys et al., 2017). Hammond et al. found populations of microglia in aged mice and in demyelinating injury with increased gene expression identical to or similar to those that define the *Cxcl10*^*hi*^ and *Ccl4*^*hi*^ TBI microglia (Hammond et al., 2019). Hammond further showed that *Cxcl10*^+^ and *Ccl4*^+^ microglia localized to and were upregulated in demyelinated lesions (Hammond et al., 2019). Jordao et al. also identified disease-associated microglia subsets in experimental autoimmune encephalitis (EAE), one of that shares defining features with *Ccl4*^*hi*^ TBI microglia (Jordão et al., 2019). Injury-associated microglia were identified in the setting of spinal cord injury (Wahane et al., 2021). Thus, microglia subset biology in neurodegeneration may be similar across neuroinflammatory states and broadly useful.

These data uncover crosstalk between macrophages and microglia, where microglia subtypes recruit monocytes to the injured CNS by expression of *Ccl2* and *Ccl3* and then differentiated monocytes/macrophages direct a type I IFN response in microglia subsets. Our study identifies circulating CCR2^+^ cells as a source influencing the type I IFN response in microglia. While type I IFN signaling in neurons and astrocytes also affects microglia activation (Chhatbar et al., 2018), the relationship between microglia and monocytes were not well-defined. Interestingly, a previous study of cell replacement therapy in a model of amyotrophic lateral sclerosis (ALS) using a treatment of NOX2-deficient monocytes ameliorated microglia activation leading to extended animal survival in ALS (Chiot et al., 2020). It would be interesting to test whether monocytes deficient in type I IFNs could be used to modulate microglia and provide benefit to TBI. Replacing and/or modulating circulating monocytes may be easier to manipulate than targeting microglia directly.

We elucidate potential mechanisms of neuroprotection related to targeting CCR2. Our initial hypothesis was that the *Ccr2*-independent macrophages in the brain would express a more proreparative phenotype. However, *Ccr2* deficiency, besides leading to a robust reduction in the migration of circulating monocytes to the injured CNS, did not yield clear or large shifts in gene expression in the macrophage/ dendritic cell subclusters. We conclude that one mechanism by which the *Ccr2*^−/−^ mice fare better after TBI is simply the reduced number of inflammatory macrophages in the brain. In CCR2-targeted mice, we also observe a reduction in Ly6C^hi^ *Chil3*^*hi*^ macrophages that were enriched in type I IFN responses, implicating the reduction of innate immune pathways that promote type I IFNs as a contributing mechanism of neuroprotection. The studies by Karve et al. found that the IFN response in TBI was detected in hematopoietic cells, supporting the hypothesis that *Ccr2*^+^ macrophages are partially responsible for the pathogenic IFN in TBI (Karve et al., 2016). Pathogenic *Cxcl10*^+^ monocytes have also been reported in EAE (Giladi et al., 2020).

We find additional mechanisms and effects of targeting *Ccr2* by examining microglia. Microglia in *Ccr2*^−/−^ TBI mice show evidence of reduced activation compared to WT TBI microglia. The absence of CCR2 significantly alter the proportion and gene-expression profile of specific microglia subsets. Our study links infiltrating monocytes to the advocation of potentially neurotoxic subtypes of microglia. There was the recurring and reproducible observation of a moderate reduction in the expression of *Cxcl10* and other ISGs in multiple microglia subsets with high statistical significance. Our data show that modulation of microglia subset phenotypes can be linked with beneficial outcomes.

The type I IFN response is a critical innate immune pathway and is relevant to human TBI. IFNβ was robustly upregulated in human brains 6 hours after TBI (Karve et al., 2016; Roselli et al., 2018). In mice, a deficiency in or blockade of IFNAR1 improved brain pathology after TBI (Karve et al., 2016). The type I IFN response can drive inflammation in AD and EAE (Roy et al., 2020; Giladi et al., 2020). Thus, a second potential mechanism by which targeting *Ccr2* is beneficial may be a consequent dampening of the type I IFN response, pointing to this pathway as an additional target for TBI therapy.

The blockade of macrophages may also reduce other harmful pathways in TBI. Microglia subsets from *Ccr2*^−/−^ TBI mice also express less *Lgals3*, which has been identified as a therapeutic target in TBI (Yip et al., 2017). Interestingly, *Ccl4*^*hi*^ microglia, the subset sharing the most markers with DAM, are blunted in cell proportions in *Ccr2*^−/−^ TBI mice. Although these were protective cells in the setting of AD, it is possible that the benefit associated with the dampening of these cells in TBI is due to reduced immune activation and/or reduced damage. CCL4 is a ligand for CCR5, a chemokine receptor on macrophages, microglia, and T cells, and CCR5 was recently shown to be a promising target to ameliorate TBI and stroke suggesting that less of these cells may be beneficial to TBI (Joy et al., 2019). *Clec7a* is also a marker for the *Ccl4*^*hi*^ TBI microglia, and it plays critical roles in neuroinflammation (Deerhake et al., 2021). Taken together, these pathways and DAM-like microglia may play an important role in TBI and further evaluation is required.

Furthermore, our preclinical studies reveal that inhibiting hCCR2 with CCX872 *in vivo* 2 h after injury reduces the type I IFN response in the acutely injured brain tissue and improves TBI outcomes. Pharmacological blockade of hCCR2 after TBI in hCCR2 knockin mice using CCX872 improved performance in cognitive behavioral testing one month after injury. CCX872 is in clinical trials for treatment of other human diseases such as pancreatic cancer and hepatic steatosis (Flores-Toro et al., 2020) and thus is translationally relevant. Importantly, our pharmacological studies to target hCCR2 indicate that benefits and changes in inflammation observed in the *Ccr2*^−/−^ mice (Hsieh et al., 2014) were not due to developmental differences in the knockout animals. CCX872 treatment in hCCR2 knock-in mice after TBI also significantly reduced expression of *Irf7*, reinforcing the link between a reduced type I IFN response and neuroprotection *in vivo*. Future studies to extend our findings to human TBI would be exciting.

## STAR★METHODS

### RESOURCE AVAILABILITY

#### Lead contact

Further information and requests for resources and reagents should be directed to and will be fulfilled by the Lead Contact, Christine L. Hsieh (christine.hsieh@ucsf.edu).

#### Materials availability

This study did not generate new unique reagents.

#### Data and code availability

Single-cell RNA-seq data have been deposited at GEO and are publicly available as of the date of publication. The accession number is listed in the key resources table. Microscopy data reported in this paper will be shared by the lead contact upon request.

All original code has been deposited at Zenodo and is publicly available as of the date of publication. The DOI is listed in the key resources table.

Any additional information required to reanalyze the data reported in this paper is available from the lead contact upon request.

### EXPERIMENTAL MODEL AND SUBJECT DETAILS

#### Animals

All mice were housed in a controlled environment (12h light/12h dark cycle, ~20°C). WT C57BL/6 male (RRID:IMSR_JAX:000664) cage mate mice (aged 12-16 weeks) were received from Jackson Laboratories (Sacramento, CA) and served as controls. *Ccr2* knockout mice (Boring et al., 1998) were backcrossed onto a C57BL/6 background for nine generations and were from Jackson Laboratories (Bar Harbor, ME). Human *Ccr2* knock-in mice were a generous gift from Israel Charo, Tim Sullivan, and James Campbell at ChemoCentryx (Mountain View, CA). CCR2 transgenic mouse breeding colonies were established and maintained at the San Francisco VA Medical Center. To reduce variability, male mice were used for this initial study. Mouse experiments were performed according to the rules and guidelines established by the Institutional Animal Care and Use Committee at the San Francisco VA Medical Center.

### METHOD DETAILS

#### Controlled cortical impact surgery and drug treatment

Controlled cortical impact (CCI) or sham surgery was performed as approved by the VA Animal Care Committee. Animals were anesthetized with 3% isoflurane with oxygen and were administered bupivacaine (4 mg/kg) subcutaneously. A midline incision across the scalp was made, and a craniectomy was performed over the right parietal cortex. The target for the impact of coordinates was 1.5 mm right lateral and 2.3 mm posterior from the Bregma point. No animals used in this study showed excessive bleeding or indication of breaching the dura during the craniectomy. For TBI animals, a circular, flat-tipped piston induced an injury at 3 m/s, 150 ms duration, with a depth of 1.5 mm (Amscien Instruments, Richmond VA, with extensive modifications by H&R Machine, Capay, CA). After the bleeding was stopped, the skin was stapled closed together. Sham-injured mice received surgical procedures without piston impact. All mice received buprenex (0.05 mg/kg up to two times/day for 24h) or sustained release buprenorphine (1 mg/kg) post-operation and 2 mL of saline s.c. to prevent dehydration.

For a subset of experiments, an antagonistic hCCR2 small molecule inhibitor (30 or 100 mg/kg) or vehicle (1% hypomethylcellulose) was administered subcutaneously beginning at 2h post-surgery (day 0), and then 1x/day daily thereafter through day 5 or until sacrifice, whichever occurred earlier. Drugs and vehicle were provided by ChemoCentryx (Mountain View, CA).

#### Brain leukocyte isolation, flow cytometry, and cell sorting

Four days after TBI, animals were euthanized and perfused through the heart with ice-cold GKN buffer (8 g/L NaCl, 0.4 g/L KCl, 1.41 g/L Na_2_HPO4, 0.6 g/L NaH_2_PO^4^, and 2 g/L D(+) glucose, pH 7.4)(Hsieh et al., 2013, Hsieh et al., 2014, Kim et al., 2016). Ipsilateral brain hemispheres were harvested and the olfactory bulbs removed. Tissues were minced and washed in cold GKN buffer. Tissue chunks were resuspended in 2.5 mL of digestion buffer (NOSE buffer (4 g/L MgCl_2_, 2.55 g/L CaCl_2_, 3.73 g/L KCl, 8.95 g/L NaCl, pH 6-7) with 200 U/ml DNase I (Sigma-Aldrich) and 0.2 mg/mL Collagenase I (Worthington Biochemical) and incubated at 37C in a dry incubator for 1h with shaking every 15 min. All samples were placed on ice to halt enzymatic activity. Tissues were crushed through a 100 micron nylon filter cup (BD Biosciences) and the cell suspension was washed with GKN buffer. An isotonic Percoll solution (90% Percoll (GE Biosciences), 10% 1.5M NaCl) was made and brought to room temperature. Cells were resuspended in 20 mL of a 1.03 g/mL isotonic Percoll solution in GKN buffer and underlayed with 10 mL of a 1.095 g/L isotonic Percoll solution in PBS. Cells were spun at ~850 g at room temperature for 20 min. Buffy layers were isolated.

Cells were blocked with 10% rat serum for 10 min on ice and then stained with the following antibodies: anti-CD45 PE-Cy5.5 (Clone 30-F11, Invitrogen), anti-Ly6G PE-eFluor610 (Clone 1A8, Invitrogen), anti-CD11b PE (Clone M1/70, BD Biosciences), anti-Ly6C PE-Cy7 (Clone AL-21, BD Biosciences), and anti-CD3 FITC (Clone 17A2, BD Biosciences). DAPI (Invitrogen) was used at 1 uM to gate out dead cells. Cells were sorted on a FACS AriaIIu (BD Biosciences) at the San Francisco VA Medical Center Flow Cytometry Core Facility.

Intracellular RNA flow cytometry was performed using PrimeFlow RNA reagents (Affymetrix)(Kim et al., 2016). Fixable viability dye eFluor506 was used to exclude dead cells. Cell surface markers were stained using antibodies against CD45 (Clone 30-F11), CD11b (Clone M1/70), Ly6G (Clone 1A8), and Ly6C (Clone AL-21). RNA probes for *Arg1, Chil3*, and *Gpnmb* were used. A DapB RNA probe, a probe for RNA of a bacterial gene, served as a negative control. Cell staining was analyzed on a FACS AriaIIu at the San Francisco VA Health Care System Flow Cytometry Core Facility. Data was analyzed using FlowJoX software (Treestar).

#### Single cell RNA sequencing

Eleven individual mice were used for scRNA-seq (3 WT TBI ipsilateral hemispheres, 3 *Ccr2*^−/−^ TBI ipsilateral hemispheres, 3 WT normal brains, and 2 *Ccr2*^−/−^ normal brains). Dissected ipsilateral hemispheres were individually sorted for CD45^+^ Ly6G^−^ live singlets using a FACSAriaIIu at the San Francisco VA Health Care System Flow Cytometry Core Facility. Single cell RNA seq was performed at the Genomics Core Facility at the Institute for Human Genetics (University of California, San Francisco) using the 10X Genomics platform with gel emulsion bead technology. Chromium Single Cell 3′ Reagent Kits v3 was used according to manufacturer protocols and each sample was run on separate lanes. Libraries were sequenced on a NovaSeq6000 at the UCSF Center for Advanced Technologies with an average of 2967 average UMI per cell.

#### Relative quantitative PCR

Perfused brain tissues were stored in RNA later at −20C. RNA was isolated using TRIzol reagent (Invitrogen) and a Kinematica homogenizer (Polytron PT 10-35 GT). Reverse transcription was performed with the iScript cDNA Synthesis Kit (Bio-Rad) using a Mastercycler EP Gradient S (Eppendorf). Quantitative PCR was run using TaqMan reagents. Primer sequences used were: Irf7-Mm00516793_g1, Tnf- Mm00443258_m1, and Gapdh- Mm99999915_g1 (FAM-MGB, ThermoFisher) as an endogenous control. PCR amplification was performed on QuantStudio 7 Flex (Applied Biosystems) at the San Francisco VA Health Care System Molecular Biology Core Facility.

#### Fluorescent Immunohistochemistry

Anesthetized mice were perfused with ice cold saline followed by 4% paraformaldehyde. Brains were post-fixed overnight in 4% paraformaldehyde and then immersed in 15% sucrose for 6h followed by 30% sucrose for 6h. Brains were embedded in Tissue-Tek optimal cutting temperature (OCT) compound (Sakura Finetech, Torrance, CA), frozen on dry ice and stored at −80°C. Brains were cut coronally into 40um thick free-floating sections into PBS. Sections were blocked for 1h with 10% donkey serum in TBS containing 3% BSA and 0.4% Triton X-100 and incubated with primary antibodies overnight. Primary antibodies were against Iba-1 (rabbit polyclonal; Wako, Richmond, VA), Cxcl10 (goat IgG, R&D Systems, Minneapolis, MN) and NeuN (clone A60, Millipore, Burlington, MA). The following secondary antibodies were used: Donkey anti-goat IgG (H+L) Cross-Adsorbed Secondary Antibody, Alexa Fluor 488; Donkey anti-rabbit IgG (H+L) Highly Cross-Adsorbed Secondary Antibody, Alexa Fluor 568; Donkey anti-mouse IgG (H+L) Highly Cross-Adsorbed Secondary Antibody, Alexa Fluor 647 (Thermo Fisher Scientific, Waltham, MA) for 2h at room temperature. DAPI (1:2000, Sigma-Aldrich, St Louis, MO) was used to identify nuclei before mounting with Fluoromount-G (Thermo Fisher Scientific, Waltham, MA). Images were acquired with a confocal laser scanning microscope (Zeiss LSM510 meta). Pictures were analyzed with Zeiss Zen microscope software. 3 animals/group were analyzed.

#### Chromogenic Immunohistochemistry

Formalin-fixed mouse brains were sectioned coronally through the middle of the visualized CCI lesion. The rest of the brain was sectioned into 2mm thick coronal sections as a 6-piece coronal trim. Pieces were placed rostrally face down, emanating from the mid-lesional section. These coronal sections were processed into FFPE blocks. Blocks were sectioned at 5μm on a DNS AS-400 Autosectioner. H&E and immunoperoxidase stains were performed (Iba1-Ki67) on Ventana Ultra IHC machines.

Stained slides were scanned at 20x using a Panoramic P250 slide scanner. Images were analyzed using Visiopharm software and custom image analysis algorithms. Ki67 and Iba1 were analyzed with a morphology algorithm to detect percent area of total immunoreactivity and morphology parameters based on detected positivity. Data were compared among CCI and sham groups using 2-factor ANOVAs.

#### Behavior studies

The cued platform version of the Morris Water Maze(Liu et al., 2007, Raber et al., 2004, Suh et al., 2005, Hong et al., 2007, Hsieh et al., 2014) was performed starting at 4 weeks post-TBI at the San Francisco VA Animal Behavior Core Facility. A swimming pool (Maze Engineers) filled with opaque water was monitored using video tracking software, Ethovision XT13 (Noldus), to analyze animals’ swim paths. A platform with a cue on top was placed in opaque water. Animals were trained to locate the platform with three trials per session for two sessions in one day. The maximum time per trial was 60 s. If an animal had not located the platform after 60 s, a handler blinded to the animal group would guide the animal to the platform.

The open field test(Hong et al., 2007, Raber et al., 2004, Suh et al., 2005, Hsieh et al., 2014) assessed animals’ spontaneous locomotor activity and baseline anxiety starting at 3 weeks post-TBI. Animals were placed in a novel environment inside a plexiglass enclosure (40 × 40 inches) surrounded by automated infrared photocells connected to a computer with KinderScientific software (Hamilton & Kinder) to record data. Beam breaks generated by movement were observed, allowing measurements of spontaneous locomotor activity. The amount of time spent in the center of the open field arena was used as an indicator of baseline anxiety. Decreased time spent in the center zone was used as an indicator of anxiety-like behavior. Animals were tested for 10 min/day for two days.

Rotarod (Hong et al., 2007, Hsieh et al., 2014, Liu et al., 2007, Raber et al., 2004, Suh et al., 2005) (TSE Systems) was used to assess motor balance and coordination in mice three weeks after TBI. Mice were placed on a rotating rod that accelerated to 40 rpm over 300 s. The length of time the mouse could stay on the rod before falling off was recorded. Animals were assessed five trials/day for two days.

### QUANTIFICATION AND STATISTICAL ANALYSIS

#### Statistical analysis

Statistical analysis of flow cytometry data was performed using Prism 7.0 & 8.0 (Graphpad). A one way ANOVA was used to determine statistical significance among groups, followed by a Kruskal-Wallis test and Dunn’s multiple comparisons test (*p < 0.05, **p < 0.01, ***p < 0.001, ****p < 0.0001).

Relative quantification PCR data was analyzed using Prism 7.0 software (Graphpad). PCR datasets per gene of interest were analyzed using ordinary one-way ANOVA and Tukey’s multiple comparisons test, with a single pooled variance.

Sample sizes can be found in figure legends, and p values can be found in the Results section and/or indicated in the figures.

#### Single-cell RNA sequencing analysis

STAR Solo v2.7.2b was used to align reads to the mouse genome (mm10) and aggregate UMI counts per gene per cell. STAR Solo count matrix outputs were then imported into Seurat and samples were combined following v3 integration methodology. Samples were aligned using 30 dimensions during integration and used for downstream analysis, including clustering and visualization with UMAP. After using the join graph-based clustering at a variety of resolutions, we settled on 20 total groups. Marker genes for each cluster were determined using Seurat’s (version 3.1.1) ‘FindMarkers’ function with the parameters logfc.threshold = 0.25, min.pct = 0.25, only.pos = T. Cell type identities were determined from cell lineage genes (increasing CC vectors and higher resolutions for clustering lead to cumbersome results, due to an extremely high number of clusters emerging before this group of cells would cluster independently). Each cluster was assigned to a cell type and subtype, and differential expression testing between wild-type and *Ccr2*^−/−^ was done with FindMarkers function utilizing the Wilcox rank-sum test (parameters logfc.threshold = 0.1, min.pct = 0.1, only.pos = F) inside each cell type and subtype. Wilcox rank-sum test p values were then corrected for multiple testing using the Bonferroni correction method based on the number genes tested. Cell type, cell subtype, and WT versus *Ccr2*^−/−^ heatmaps were generated from top 10 (or fewer if there were not 10) differentially expressed gene lists using Complex Heatmap. R package 3.6.1 was used for bioinformatics analyses. Other attached R packages used were: limma_3.40.6, scatterpie_0.1.4, Formula_1.2-3, data.table_1.12.6, Seurat_3.1.1, scales_1.0.0, ggrepel_0.8.1, Matrix_1.2-17, reshape2_1.4.3, cowplot_1.0.0, RColorBrewer_1.1-2, viridis_0.5.1, viridisLite_0.3.0, dplyr_0.8.3, ggplot2_3.2.1, future_1.14.0 (https://cran.r-project.org/).

Statistical analysis found significant differences in cell numbers in microglia and in the macrophage/dendritic cell clusters by using two-way ANOVAs followed by Tukey’s multiple comparisons tests using Prism 8.0.

#### Fluorescent immunohistochemistry analysis

Fluorescent images were captured using a Leica SP5 laser scanning confocal microscope at the UCSF Biological Imaging Development CoLab. Quantification of IBA1^+^CXCL10^+^ microglia/macrophages was performed using the Zeiss LSM510META confocal microscope and Zen microscope software. Three animals/group were analyzed. Unpaired t tests with Holm-Sidak correction were performed post hoc.

#### Chromogenic immunohistochemistry

This protocol was performed on the Ventana Discovery ULTRA with heat induced antigen retrieval in Cell Conditioner 1 buffer (Tris-based EDTA). The Ki67 antibody was diluted 1:50, and the Iba1 antibody was diluted 1:4000 (0.125 ug/ml working concentration). Slides were analyzed for staining with anti-Ki67 and anti-IBA1 antibodies using Visiopharm software. Ki67 and IBA1 staining were analyzed with a morphology algorithm to detect percent area of total immunoreactivity and morphology parameters based on detected positivity. Data was compared among TBI and sham groups utilizing two-way ANOVAs to assess for statistical significance, followed by an unpaired t test with Benjamini, Krieger, Yekutieli correction.

#### Behavior studies statistical analysis

For Morris Water Maze, TBI groups (n = 17/group) and sham groups (n = 4-9) were analyzed. Rank summary scores(Possin et al., 2016) followed by a one way analysis of variance (ANOVA) (Prism 7.0, Graphpad) were used for evaluation of statistical significance.

We analyzed open field and rotor rod performance in TBI (n = 17-18/group) and sham (n = 7-9/group) groups. Two-way ANOVAs were used to assess statistical significance on each day of the open field test, followed by Tukey’s multiple comparisons test.

The animals’ average per day was used for quantitation of the rotor rod. Two-way ANOVAs were used to assess statistical significance at each time point, followed by Tukey’s multiple comparisons test.

## Supplementary Material

1

## Figures and Tables

**Table:** KEY RESOURCES TABLE

Reagent or resource	Source	Identifier
Antibodies		
Mouse monoclonal anti-CD45 PE	Invitrogen	Cat# 12-0451-82; RRID:AB_465668; Clone 30-F11
Mouse monoclonal anti-Ly6G PE-eFluor610	Invitrogen	Cat# 61-9668-82; RRID:AB_2574679; Clone 1A8
Mouse monoclonal anti-CD45 PE-Cy5.5	Invitrogen	Cat# 35-0451-82; RRID:AB_469718; Clone 30-F11
Mouse monoclonal anti-CD11b PE	BD Biosciences	Cat# 557397; RRID:AB_396680; Clone M1/70
Mouse monoclonal anti-Ly6C PE-Cy7	BD Biosciences	Cat# 560593; RRID:AB_1727557; Clone Al-21
Mouse monoclonal anti-CD3 FITC	BD Biosciences	Cat# 561798; RRID:AB_10898341; Clone 17A2
Rabbit polyclonal anti-Iba-1	Wako	Cat# 019-19741; RRID:AB_839504
Goat IgG anti-Cxcl10	R&D Systems	Cat# AF-466-NA; RRID:AB_2292487
Mouse monoclonal anti-NeuN	Millipore	Cat# MAB377; RRID:AB_2298772; Clone A60
Donkey anti-goat IgG (H+L) Cross-Adsorbed Secondary Antibody, Alexa Fluor 488	ThermoFisher	Cat# A-11055, RRID:AB_2534102
Donkey anti-mouse IgG (H+L) Highly Cross-Adsorbed Secondary Antibody, Alexa Fluor 647	ThermoFisher	Cat# A-31571, RRID:AB_162542
Donkey anti-rabbit IgG (H+L) Highly Cross-Adsorbed Secondary Antibody, Alexa Fluor 568	ThermoFisher	Cat# A10042, RRID:AB_2534017
RNA probe: Mouse Arg1 Type 1	Affymetrix	Ref# VB1-17389
RNA probe: Mouse Gpnmb Type 1	Affymetrix	Ref# VB1-17176
RNA probe: Mouse Dapb Type 1	Affymetrix	Ref# VF1-11712
RNA probe: Mouse Chil3 Type 1	Affymetrix	Ref# VB1-17412
Rabbit anti-mouse/human/rat Iba1 pAb	Wako	Cat# 019-19741; RRID:AB_839504
Rabbit anti-Ki67 monoclonal antibody	ThermoScientific	Cat# RM-9106-S; RRID:AB_149707
Chemicals, peptides, and recombinant proteins		
Human CCR2 small molecule inhibitor, CCX872	ChemoCentryx	N/A
1% hypomethylcellulose (vehicle)	ChemoCentryx	N/A
Critical commercial assays		
PrimeFlow RNA Assay	Affymetrix	Cat# 19361
Chromium Single Cell 3′ Reagent Kits v2	10x Genomics	Cat# PN-120237
TruSeq Stranded mRNA Library Prep	Illumina	Cat# 20020595
iScript cDNA Synthesis Kit	Bio-Rad	Cat# 1708891
Deposited data		
Single Cell RNA Sequencing Data	https://www.ncbi.nlm.nih.gov/geo/	GEO: GSE175430
Original code	https://zenodo.org/record/5178424	https://doi.org/10.5281/zenodo.5178424
Experimental models: Organisms/strains		
C57BL6/J	Jackson Laboratories	RRID:IMSR_JAX:000664
*Ccr2*^−/−^ mice	Jackson Laboratories	N/A
Human *Ccr2* knock-in mice	ChemoCentryx	N/A
Oligonucleotides		
Cxcl10 TaqMan Assay (FAM-MGB)	ThermoFisher	Cat # 4331182; Assay ID: Mm00445235_m1
Irf7 TaqMan Assay (FAM-MGB)	ThermoFisher	Cat # 4331182; Assay ID: Mm00516793_g1
Tnf Taqman Assay (FAM-MGB)	ThermoFisher	Cat # 4331182; Assay ID: Mm00443258_m1
Gapdh TaqMan Assay (FAM-MGB)	ThermoFisher	Cat # 4331182; Assay ID: Mm99999915_g1
Software and algorithms		
FlowJo v10	BD	RRID:SCR_008520; https://www.flowjo.com/solutions/flowjo
Cellranger	10x Genomics	https://support.10xgenomics.com/single-cell-gene-expression/software/pipelines/latest/using/count
STARsolo	Alex Dobin, dobin@cshl.edu	https://github.com/alexdobin/STAR/blob/master/docs/STARsolo.md
	https://groups.google.com/g/ma-star	
R 3.6.1		https://cran.r-project.org/
Seurat 3.1.1		RRID:SCR_007322; http://seurat.r-forge.r-project.org/
Prism 7.0 and 8.0	GraphPad	RRID:SCR_002798; https://www.graphpad.com:443/
Ethovision XT 13	Noldus	RRID:SCR_000441
Motor Monitor	Kinder Scientific	N/A

## Highlights

- TBI elevates distinct phenotypes of microglia, macrophages, and dendritic cells
- *Ccr2* deficiency alters cell proportions and reduces ISG expression in microglia
- TBI induces crosstalk between microglia and circulating monocytes
- Preclinical translational studies to target human CCR2 after TBI improves outcomes

## References

[R1] Becht E, McInnes L, Healy J, Dutertre CA, Kwok IWH, Ng LG, Ginhoux F, and Newell EW (2018). Dimensionality reduction for visualizing single-cell data using UMAP. Nat. Biotechnol Published online 12 3, 2018. 10.1038/nbt.4314. 30531897

[R2] Bennett ML, Bennett FC, Liddelow SA, Ajami B, Zamanian JL, Fernhoff NB, Mulinyawe SB, Bohlen CJ, Adil A, Tucker A, (2016). New tools for studying microglia in the mouse and human CNS. Proc. Natl. Acad. Sci. USA 113, E1738–E1746. 26884166 10.1073/pnas.1525528113 PMC4812770

[R3] Boring L, Gosling J, Cleary M, and Charo IF (1998). Decreased lesion formation in CCR2−/− mice reveals a role for chemokines in the initiation of atherosclerosis. Nature 394, 894–897. 9732872 10.1038/29788

[R4] Butler A, Hoffman P, Smibert P, Papalexi E, and Satija R (2018). Integrating single-cell transcriptomic data across different conditions, technologies, and species. Nat. Biotechnol 36, 411–420. 29608179 10.1038/nbt.4096 PMC6700744

[R5] Buttgereit A, Lelios I, Yu X, Vrohlings M, Krakoski NR, Gautier EL, Nishinakamura R, Becher B, and Greter M (2016). Sall1 is a transcriptional regulator defining microglia identity and function. Nat. Immunol 17, 1397–1406. 27776109 10.1038/ni.3585

[R6] Chawla A (2010). Control of macrophage activation and function by PPARs. Circ. Res 106, 1559–1569. 20508200 10.1161/CIRCRESAHA.110.216523 PMC2897247

[R7] Chen X, Wang Y, Nelson D, Tian S, Mulvey E, Patel B, Conti I, Jaen J, and Rollins BJ (2016). CCL2/CCR2 Regulates the Tumor Microenvironment in HER-2/neu-Driven Mammary Carcinomas in Mice. PLoS ONE 11, e0165595. 27820834 10.1371/journal.pone.0165595 PMC5098736

[R8] Cheon H, Borden EC, and Stark GR (2014). Interferons and their stimulated genes in the tumor microenvironment. Semin. Oncol 41, 156–173. 24787290 10.1053/j.seminoncol.2014.02.002 PMC4118773

[R9] Chhatbar C, Detje CN, Grabski E, Borst K, Spanier J, Ghita L, Elliott DA, Jordão MJC, Mueller N, Sutton J, (2018). Type I Interferon Receptor Signaling of Neurons and Astrocytes Regulates Microglia Activation during Viral Encephalitis. Cell Rep. 25, 118–129. 30282022 10.1016/j.celrep.2018.09.003 PMC7103936

[R10] Chiot A, Zaïdi S, Iltis C, Ribon M, Berriat F, Schiaffino L, Jolly A, de la Grange P, Mallat M, Bohl D, (2020). Modifying macrophages at the periphery has the capacity to change microglial reactivity and to extend ALS survival. Nat. Neurosci 23, 1339–1351. 33077946 10.1038/s41593-020-00718-z

[R11] Corps KN, Roth TL, and McGavern DB (2015). Inflammation and neuroprotection in traumatic brain injury. JAMA Neurol. 72, 355–362. 25599342 10.1001/jamaneurol.2014.3558 PMC5001842

[R12] Deerhake ME, Danzaki K, Inoue M, Cardakli ED, Nonaka T, Aggarwal N, Barclay WE, Ji RR, and Shinohara ML (2021). Dectin-1 limits autoimmune neuroinflammation and promotes myeloid cell-astrocyte crosstalk via Card9-independent expression of Oncostatin M. Immunity 54, 484–498. 33581044 10.1016/j.immuni.2021.01.004 PMC7956124

[R13] Elder GA (2015). Update on TBI and Cognitive Impairment in Military Veterans. Curr. Neurol. Neurosci. Rep 15, 68. 26299275 10.1007/s11910-015-0591-8

[R14] Ertürk A, Mentz S, Stout EE, Hedehus M, Dominguez SL, Neumaier L, Krammer F, Llovera G, Srinivasan K, Hansen DV, (2016). Interfering with the Chronic Immune Response Rescues Chronic Degeneration After Traumatic Brain Injury. J. Neurosci 36, 9962–9975. 27656033 10.1523/JNEUROSCI.1898-15.2016 PMC6705567

[R15] Flores-Toro JA, Luo D, Gopinath A, Sarkisian MR, Campbell JJ, Charo IF, Singh R, Schall TJ, Datta M, Jain RK, (2020). CCR2 inhibition reduces tumor myeloid cells and unmasks a checkpoint inhibitor effect to slow progression of resistant murine gliomas. Proc. Natl. Acad. Sci. USA 117, 1129–1138. 31879345 10.1073/pnas.1910856117 PMC6969504

[R16] Giladi A, Wagner LK, Li H, Dörr D, Medaglia C, Paul F, Shemer A, Jung S, Yona S, Mack M, (2020). Cxcl10^+^ monocytes define a pathogenic subset in the central nervous system during autoimmune neuroinflammation. Nat. Immunol 21, 525–534. 32313246 10.1038/s41590-020-0661-1

[R17] Hammond TR, Dufort C, Dissing-Olesen L, Giera S, Young A, Wysoker A, Walker AJ, Gergits F, Segel M, Nemesh J, (2019). Single-Cell RNA Sequencing of Microglia throughout the Mouse Lifespan and in the Injured Brain Reveals Complex Cell-State Changes. Immunity 50, 253–271. 30471926 10.1016/j.immuni.2018.11.004 PMC6655561

[R18] Hinson HE, Rowell S, and Schreiber M (2015). Clinical evidence of inflammation driving secondary brain injury: a systematic review. J. Trauma Acute Care Surg 78, 184–191. 25539220 10.1097/TA.0000000000000468 PMC4297199

[R19] Hong SM, Liu Z, Fan Y, Neumann M, Won SJ, Lac D, Lum X, Weinstein PR, and Liu J (2007). Reduced hippocampal neurogenesis and skill reaching performance in adult Emx1 mutant mice. Exp. Neurol 206, 24–32. 17490651 10.1016/j.expneurol.2007.03.028

[R20] Hsieh CL, Kim CC, Ryba BE, Niemi EC, Bando JK, Locksley RM, Liu J, Nakamura MC, and Seaman WE (2013). Traumatic brain injury induces macrophage subsets in the brain. Eur. J. Immunol 43, 2010–2022. 23630120 10.1002/eji.201243084 PMC4210355

[R21] Hsieh CL, Niemi EC, Wang SH, Lee CC, Bingham D, Zhang J, Cozen ML, Charo I, Huang EJ, Liu J, and Nakamura MC (2014). CCR2 deficiency impairs macrophage infiltration and improves cognitive function after traumatic brain injury. J. Neurotrauma 31, 1677–1688. 24806994 10.1089/neu.2013.3252 PMC4545982

[R22] Israelsson C, Bengtsson H, Lobell A, Nilsson LN, Kylberg A, Isaksson M, Wootz H, Lannfelt L, Kullander K, Hillered L, and Ebendal T (2010). Appearance of Cxcl10-expressing cell clusters is common for traumatic brain injury and neurodegenerative disorders. Eur. J. Neurosci 31, 852–863. 20374285 10.1111/j.1460-9568.2010.07105.x

[R23] Ivashkiv LB, and Donlin LT (2014). Regulation of type I interferon responses. Nat. Rev. Immunol 14, 36–49. 24362405 10.1038/nri3581 PMC4084561

[R24] Jaitin DA, Adlung L, Thaiss CA, Weiner A, Li B, Descamps H, Lundgren P, Bleriot C, Liu Z, Deczkowska A, (2019). Lipid-Associated Macrophages Control Metabolic Homeostasis in a Trem2-Dependent Manner. Cell 178, 686–698.e14. 31257031 10.1016/j.cell.2019.05.054 PMC7068689

[R25] Jassam YN, Izzy S, Whalen M, McGavern DB, and El Khoury J (2017). Neuroimmunology of Traumatic Brain Injury: Time for a Paradigm Shift. Neuron 95, 1246–1265. 28910616 10.1016/j.neuron.2017.07.010 PMC5678753

[R26] Johnson VE, Stewart JE, Begbie FD, Trojanowski JQ, Smith DH, and Stewart W (2013). Inflammation and white matter degeneration persist for years after a single traumatic brain injury. Brain 136, 28–42. 23365092 10.1093/brain/aws322 PMC3562078

[R27] Jordão MJC, Sankowski R, Brendecke SM, Locatelli, G. Sagar, Tai YH, Tay TL, Schramm E, Armbruster S, Hagemeyer N, (2019). Single-cell profiling identifies myeloid cell subsets with distinct fates during neuroinflammation. Science 363, 363. 10.1126/science.aat7554 30679343

[R28] Joy MT, Ben Assayag E, Shabashov-Stone D, Liraz-Zaltsman S, Mazzitelli J, Arenas M, Abduljawad N, Kliper E, Korczyn AD, Thareja NS, (2019). CCR5 Is a Therapeutic Target for Recovery after Stroke and Traumatic Brain Injury. Cell 176, 1143–1157. 30794775 10.1016/j.cell.2019.01.044 PMC7259116

[R29] Karve IP, Zhang M, Habgood M, Frugier T, Brody KM, Sashindranath M, Ek CJ, Chappaz S, Kile BT, Wright D, (2016). Ablation of Type-1 IFN Signaling in Hematopoietic Cells Confers Protection Following Traumatic Brain Injury. eNeuro 3, ENEURO.0128-15.2016. 10.1523/ENEURO.0128-15.2016 PMC4757777 27022620

[R30] Keren-Shaul H, Spinrad A, Weiner A, Matcovitch-Natan O, Dvir-Szternfeld R, Ulland TK, David E, Baruch K, Lara-Astaiso D, Toth B, (2017). A Unique Microglia Type Associated with Restricting Development of Alzheimer’s Disease. Cell 169, 1276–1290. 28602351 10.1016/j.cell.2017.05.018

[R31] Kim CC, Nakamura MC, and Hsieh CL (2016). Brain trauma elicits non-canonical macrophage activation states. J. Neuroinflammation 13, 117. 27220367 10.1186/s12974-016-0581-z PMC4879757

[R32] Li Q, Cheng Z, Zhou L, Darmanis S, Neff NF, Okamoto J, Gulati G, Bennett ML, Sun LO, Clarke LE, (2019). Developmental Heterogeneity of Microglia and Brain Myeloid Cells Revealed by Deep Single-Cell RNA Sequencing. Neuron 101, 207–223. 30606613 10.1016/j.neuron.2018.12.006 PMC6336504

[R33] Liu Z, Fan Y, Won SJ, Neumann M, Hu D, Zhou L, Weinstein PR, and Liu J (2007). Chronic treatment with minocycline preserves adult new neurons and reduces functional impairment after focal cerebral ischemia. Stroke 38, 146–152. 17122429 10.1161/01.STR.0000251791.64910.cd

[R34] Liu C, Wu C, Yang Q, Gao J, Li L, Yang D, and Luo L (2016). Macrophages Mediate the Repair of Brain Vascular Rupture through Direct Physical Adhesion and Mechanical Traction. Immunity 44, 1162–1176. 27156384 10.1016/j.immuni.2016.03.008

[R35] Loane DJ, Kumar A, Stoica BA, Cabatbat R, and Faden AI (2014). Progressive neurodegeneration after experimental brain trauma: association with chronic microglial activation. J. Neuropathol. Exp. Neurol 73, 14–29. 24335533 10.1097/NEN.0000000000000021 PMC4267248

[R36] Loke P, Nair MG, Parkinson J, Guiliano D, Blaxter M, and Allen JE (2002). IL-4 dependent alternatively-activated macrophages have a distinctive in vivo gene expression phenotype. BMC Immunol. 3, 7. 12098359 10.1186/1471-2172-3-7 PMC117781

[R37] Mahata B, Zhang X, Kolodziejczyk AA, Proserpio V, Haim-Vilmovsky L, Taylor AE, Hebenstreit D, Dingler FA, Moignard V, Göttgens B, (2014). Single-cell RNA sequencing reveals T helper cells synthesizing steroids de novo to contribute to immune homeostasis. Cell Rep. 7, 1130–1142. 24813893 10.1016/j.celrep.2014.04.011 PMC4039991

[R38] Martin P, and Leibovich SJ (2005). Inflammatory cells during wound repair: the good, the bad and the ugly. Trends Cell Biol. 15, 599–607. 16202600 10.1016/j.tcb.2005.09.002

[R39] Mathys H, Adaikkan C, Gao F, Young JZ, Manet E, Hemberg M, De Jager PL, Ransohoff RM, Regev A, and Tsai LH (2017). Temporal Tracking of Microglia Activation in Neurodegeneration at Single-Cell Resolution. Cell Rep. 21, 366–380. 29020624 10.1016/j.celrep.2017.09.039 PMC5642107

[R40] Merad M, Sathe P, Helft J, Miller J, and Mortha A (2013). The dendritic cell lineage: ontogeny and function of dendritic cells and their subsets in the steady state and the inflamed setting. Annu. Rev. Immunol 31, 563–604. 23516985 10.1146/annurev-immunol-020711-074950 PMC3853342

[R41] Miao Z, Ertl LS, Newland D, Zhao B, Wang Y, Zang X, Campbell JJ, Liu X, Dang T, Miao S, (2018). CCR2 antagonism leads to marked reduction in proteinuria and glomerular injury in murine models of focal segmental glomerulosclerosis (FSGS). PLoS ONE 13, e0192405. 29561839 10.1371/journal.pone.0192405 PMC5862408

[R42] Mizutani M, Pino PA, Saederup N, Charo IF, Ransohoff RM, and Cardona AE (2012). The fractalkine receptor but not CCR2 is present on microglia from embryonic development throughout adulthood. J. Immunol 188, 29–36. 22079990 10.4049/jimmunol.1100421 PMC3244524

[R43] Morganti JM, Jopson TD, Liu S, Riparip LK, Guandique CK, Gupta N, Ferguson AR, and Rosi S (2015). CCR2 antagonism alters brain macrophage polarization and ameliorates cognitive dysfunction induced by traumatic brain injury. J. Neurosci 35, 748–760. 25589768 10.1523/JNEUROSCI.2405-14.2015 PMC4293420

[R44] Murray PJ, Allen JE, Biswas SK, Fisher EA, Gilroy DW, Goerdt S, Gordon S, Hamilton JA, Ivashkiv LB, Lawrence T, (2014). Macrophage activation and polarization: nomenclature and experimental guidelines. Immunity 41, 14–20. 25035950 10.1016/j.immuni.2014.06.008 PMC4123412

[R45] Patel AP, Tirosh I, Trombetta JJ, Shalek AK, Gillespie SM, Wakimoto H, Cahill DP, Nahed BV, Curry WT, Martuza RL, (2014). Single-cell RNA-seq highlights intratumoral heterogeneity in primary glioblastoma. Science 344, 1396–1401. 24925914 10.1126/science.1254257 PMC4123637

[R46] Patil SS, Sunyer B, Höger H, and Lubec G (2009). Evaluation of spatial memory of C57BL/6J and CD1 mice in the Barnes maze, the Multiple T-maze and in the Morris water maze. Behav. Brain Res 198, 58–68. 19022298 10.1016/j.bbr.2008.10.029

[R47] Possin KL, Sanchez PE, Anderson-Bergman C, Fernandez R, Kerchner GA, Johnson ET, Davis A, Lo I, Bott NT, Kiely T, (2016). Cross-species translation of the Morris maze for Alzheimer’s disease. J. Clin. Invest 126, 779–783. 26784542 10.1172/JCI78464 PMC4731157

[R48] Potts MB, Koh SE, Whetstone WD, Walker BA, Yoneyama T, Claus CP, Manvelyan HM, and Noble-Haeusslein LJ (2006). Traumatic injury to the immature brain: inflammation, oxidative injury, and iron-mediated damage as potential therapeutic targets. NeuroRx 3, 143–153. 16554253 10.1016/j.nurx.2006.01.006 PMC3593438

[R49] Raber J, Fan Y, Matsumori Y, Liu Z, Weinstein PR, Fike JR, and Liu J (2004). Irradiation attenuates neurogenesis and exacerbates ischemia-induced deficits. Ann. Neurol 55, 381–389. 14991816 10.1002/ana.10853

[R50] Ransohoff RM (2016). A polarizing question: do M1 and M2 microglia exist? Nat. Neurosci 19, 987–991. 27459405 10.1038/nn.4338

[R51] Ricardo-Gonzalez RR, Van Dyken SJ, Schneider C, Lee J, Nussbaum JC, Liang HE, Vaka D, Eckalbar WL, Molofsky AB, Erle DJ, and Locksley RM (2018). Tissue signals imprint ILC2 identity with anticipatory function. Nat. Immunol 19, 1093–1099. 30201992 10.1038/s41590-018-0201-4 PMC6202223

[R52] Roselli F, Chandrasekar A, and Morganti-Kossmann MC (2018). Interferons in Traumatic Brain and Spinal Cord Injury: Current Evidence for Translational Application. Front. Neurol 9, 458. 29971040 10.3389/fneur.2018.00458 PMC6018073

[R53] Roy ER, Wang B, Wan YW, Chiu G, Cole A, Yin Z, Propson NE, Xu Y, Jankowsky JL, Liu Z, (2020). Type I interferon response drives neuroinflammation and synapse loss in Alzheimer disease. J. Clin. Invest 130, 1912–1930. 31917687 10.1172/JCI133737 PMC7108898

[R54] Russo MV, Latour LL, and McGavern DB (2018). Distinct myeloid cell subsets promote meningeal remodeling and vascular repair after mild traumatic brain injury. Nat. Immunol 19, 442–452. 29662169 10.1038/s41590-018-0086-2 PMC6426637

[R55] Sala Frigerio C, Wolfs L, Fattorelli N, Thrupp N, Voytyuk I, Schmidt I, Mancuso R, Chen WT, Woodbury ME, Srivastava G, (2019). The Major Risk Factors for Alzheimer’s Disease: Age, Sex, and Genes Modulate the Microglia Response to Aβ Plaques. Cell Rep. 27, 1293–1306. 31018141 10.1016/j.celrep.2019.03.099 PMC7340153

[R56] Schneider WM, Chevillotte MD, and Rice CM (2014). Interferon-stimulated genes: a complex web of host defenses. Annu. Rev. Immunol 32, 513–545. 24555472 10.1146/annurev-immunol-032713-120231 PMC4313732

[R57] Semple BD, Bye N, Rancan M, Ziebell JM, and Morganti-Kossmann MC (2010). Role of CCL2 (MCP-1) in traumatic brain injury (TBI): evidence from severe TBI patients and CCL2−/− mice. J. Cereb. Blood Flow Metab 30, 769–782. 20029451 10.1038/jcbfm.2009.262 PMC2949175

[R58] Shalek AK, Satija R, Shuga J, Trombetta JJ, Gennert D, Lu D, Chen P, Gertner RS, Gaublomme JT, Yosef N, (2014). Single-cell RNA-seq reveals dynamic paracrine control of cellular variation. Nature 510, 363–369. 24919153 10.1038/nature13437 PMC4193940

[R59] Shi C, and Pamer EG (2011). Monocyte recruitment during infection and inflammation. Nat. Rev. Immunol 11, 762–774. 21984070 10.1038/nri3070 PMC3947780

[R60] Suh SW, Aoyama K, Matsumori Y, Liu J, and Swanson RA (2005). Pyruvate administered after severe hypoglycemia reduces neuronal death and cognitive impairment. Diabetes 54, 1452–1458. 15855333 10.2337/diabetes.54.5.1452

[R61] Sullivan T, Miao Z, Dairaghi DJ, Krasinski A, Wang Y, Zhao BN, Baumgart T, Ertl LS, Pennell A, Seitz L, (2013). CCR2 antagonist CCX140-B provides renal and glycemic benefits in diabetic transgenic human CCR2 knockin mice. Am. J. Physiol. Renal Physiol 305, F1288–F1297. 23986513 10.1152/ajprenal.00316.2013 PMC4073927

[R62] Swirski FK, and Nahrendorf M (2018). Cardioimmunology: the immune system in cardiac homeostasis and disease. Nat. Rev. Immunol 18, 733–744. 30228378 10.1038/s41577-018-0065-8

[R63] Taylor CA, Bell JM, Breiding MJ, and Xu L (2017). Traumatic Brain Injury-Related Emergency Department Visits, Hospitalizations, and Deaths - United States, 2007 and 2013. MMWR Surveill. Summ 66, 1–16. 10.15585/mmwr.ss6609a1 PMC5829835 28301451

[R64] Trapnell C (2015). Defining cell types and states with single-cell genomics. Genome Res. 25, 1491–1498. 26430159 10.1101/gr.190595.115 PMC4579334

[R65] Vorhees CV, and Williams MT (2006). Morris water maze: procedures for assessing spatial and related forms of learning and memory. Nat. Protoc 1, 848–858. 17406317 10.1038/nprot.2006.116 PMC2895266

[R66] Wahane S, Zhou X, Zhou X, Guo L, Friedl MS, Kluge M, Ramakrishnan A, Shen L, Friedel CC, Zhang B, (2021). Diversified transcriptional responses of myeloid and glial cells in spinal cord injury shaped by HDAC3 activity. Sci. Adv 7, Published online 2 26, 2021. 10.1126/sciadv.abd8811. PMC7909890 33637528

[R67] Wu AR, Neff NF, Kalisky T, Dalerba P, Treutlein B, Rothenberg ME, Mburu FM, Mantalas GL, Sim S, Clarke MF, and Quake SR (2014). Quantitative assessment of single-cell RNA-sequencing methods. Nat. Methods 11, 41–46. 24141493 10.1038/nmeth.2694 PMC4022966

[R68] Xiong Y, Mahmood A, and Chopp M (2013). Animal models of traumatic brain injury. Nat. Rev. Neurosci 14, 128–142. 23329160 10.1038/nrn3407 PMC3951995

[R69] Yip PK, Carrillo-Jimenez A, King P, Vilalta A, Nomura K, Chau CC, Egerton AM, Liu ZH, Shetty AJ, Tremoleda JL, (2017). Galectin-3 released in response to traumatic brain injury acts as an alarmin orchestrating brain immune response and promoting neurodegeneration. Sci. Rep 7, 41689. 28128358 10.1038/srep41689 PMC5269662

