# Supplementary material for: CCR2 deficiency alters activation of microglia subsets in traumatic brain injury
Source: Cell Rep. Author manuscript; Available in PMC 2021 Nov 16. (PMC8594931; doi:10.1016/j.celrep.2021.109727)
Supplement: 1 [file NIHMS1746913-supplement-1.pdf]

Cell Reports, Volume *36*

Supplemental information

CCR2 deficiency alters activation

# of microglia subsets in traumatic brain injury

Kerri Somebang, Joshua Rudolph, Isabella Imhof, Luyi Li, Erene C. Niemi, Judy Shigenaga, Huy Tran, T. Michael Gill, Iris Lo, Brian A. Zabel, Gabriela Schmajuk, Brian T. Wipke, Stefka Gyoneva, Luke Jandreski, Michael Craft, Gina Benedetto, Edward D. Plowey, Israel Charo, James Campbell, Chun Jimmie Ye, S. Scott Panter, Mary C. Nakamura, Walter Eckalbar, and Christine L. Hsieh

Supplementary Figure 1

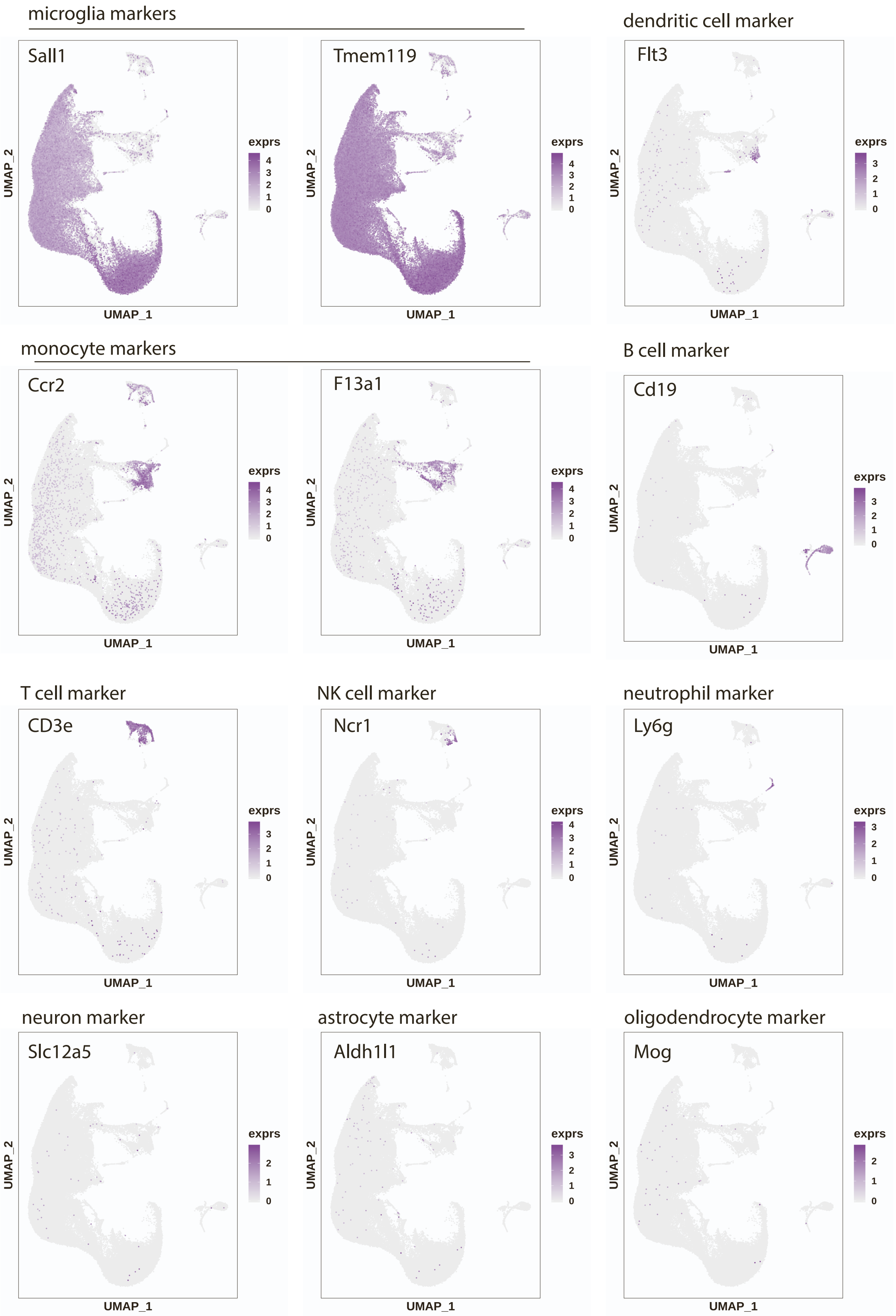

Supplementary Figure 1: Gene expression of cell lineage markers shown in UMAPs of acute TBI and normal brain white cells combined, Related to Figure 1. 111,717 cells from 11 individual adult mouse brains (n=3 WT TBI, n=3 *Ccr2*$^{-/-}$ TBI, n=3 WT normal, n=2 *Ccr2*$^{-/-}$ normal) were analyzed for their gene expression of cell lineage markers by single-cell RNA sequencing. Top row: Microglia markers include *Sall1* and *Tmem119.* Dendritic cell markers include *Flt3*. Second row: Monocyte/Macrophage DEGs include *Ccr2* and *F13a1*. B cell markers include *Cd19.* Third row: Lymphocyte genes used to define T cells include *Cd3e*, and to define NK cells we used *Ncr1* (NKp46). *Ly6g* was one of the neutrophil markers. Fourth row: Neuronal cell markers and macroglia genes include potassium-chloride transporter member 5 (*Slc12a5*), aldehyde dehydrogenase-1 (*Aldh1l1*) for astrocytes, and myelin oligodendrocyte glycoprotein (*Mog*).

Supplementary Figure 2

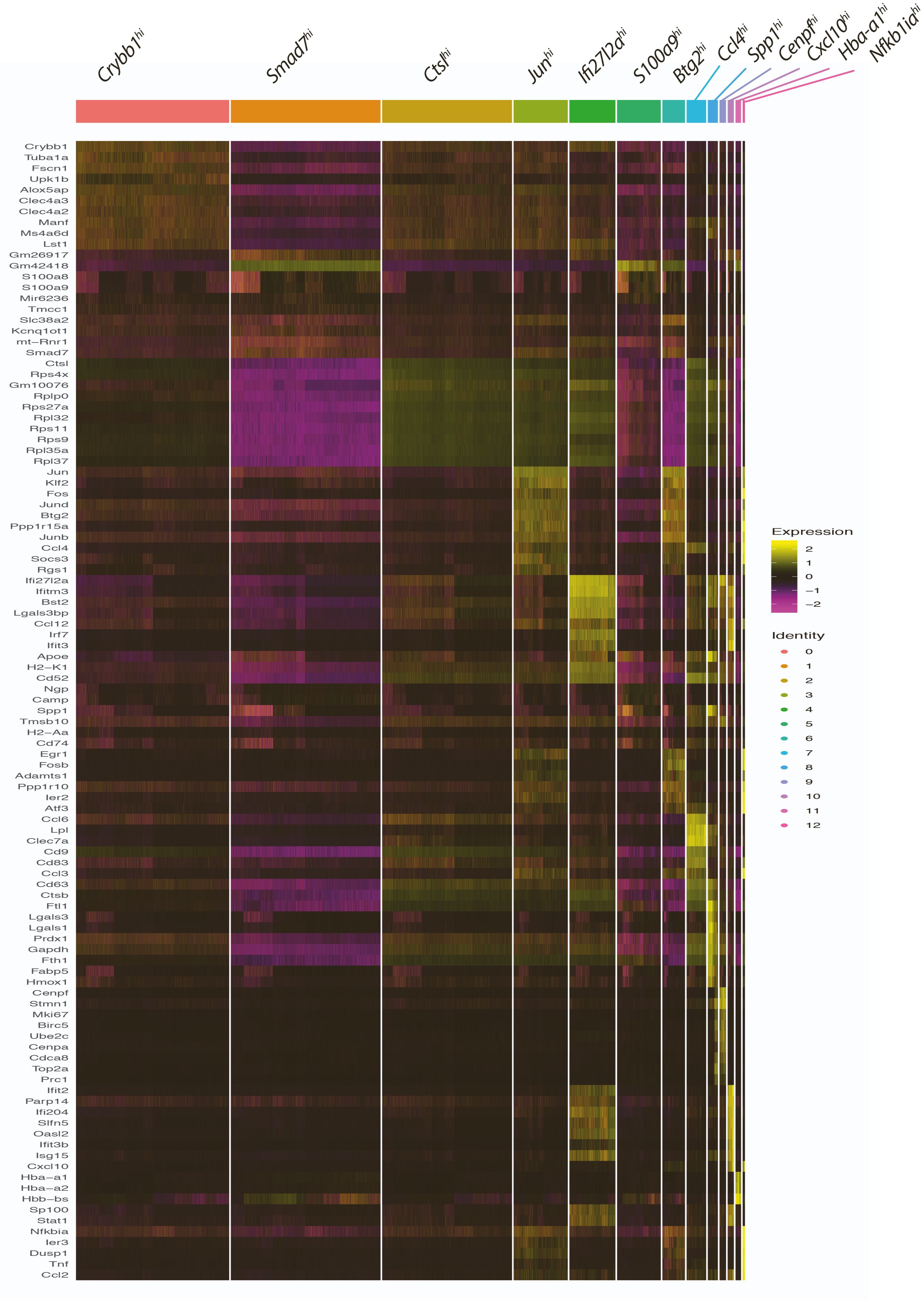

Supplementary Figure 2: Relative expression of significant DEGs defining microglia subclusters in the TBI and normal mouse brain, Related to Figure 2. A heatmap showing relative expression of the top 10 differentially expressed genes between 13 microglia subsets identified by scRNA seq. Data represent an analysis of 102,997 microglia from 11 individual acute TBI and normal mouse brains.

Supplementary Figure 3

A

	Microglia Subcluster	Enriched biological processes	q value	#Genes in set	Top DEGs (Fold Change)
1	***Cxcl10***^{hi}	• response to IFN-beta<br>• defense response to virus<br>• response to IFN-gamma<br>• response to IFN-alpha	2.0E-07<br>2.0E-07<br>2.0E-07<br>2.0E-07	20<br>25<br>13<br>8	*Ifit3* (6), *Cxcl10* (6), *Ifit2* (5), *Ccl12* (5), *Isg15* (4), *Ifit3b* (3), *Irf7* (3)
2	***Cenpf***^{hi}	• chromosome segregation<br>• mitotic nuclear division<br>• regulation of mitotic cell cycle	1.0E-06<br>1.0E-06<br>1.0E-06	25<br>22<br>28	*Cenpf* (3), *Ifi27l2a* (3), *Top2a* (3), *Mki67* (3)
3	***Ccl4***^{hi}	• leukocyte migration<br>• myeloid leukocyte migration<br>• response to IFN-gamma<br>• granulocyte chemotaxis	3.0E-06<br>3.0E-06<br>3.0E-06<br>3.0E-06	17<br>13<br>11<br>10	*Ccl4* (3), *Ccl6* (3), *Lpl* (3), *Clec7a* (3), *Cd9* (2), *Cd52* (2), *Cd83* (2), *Ccl3* (2), *Cd63* (2), *Ctsb* (2), *Ank* (2), *Ctsl* (2), *Cst7* (2), *Ctsd* (2), *Apoe* (1.6)
4	***Ifi27l2a*** ^{hi}	• response to virus<br>• response to IFN-beta<br>• antigen processing & presentation	2.0E-04<br>2.0E-04<br>2.0E-04	16<br>9<br>8	*Ifi27l2a* (3), *Ifitm3* (3), *Irf7* (2), *Bst2* (2), *Lgals3bp* (2)
5	***Spp1***^{hi}	• response to IFN-gamma<br>• leukocyte chemotaxis	1.0E-05<br>2.0E-05	12<br>13	*Spp1* (9), *Apoe* (4), *Flt1* (4), *Hmox1* (3)
6	***Btg2***^{hi}	• regulation of hemopoiesis<br>• leukocyte cell-cell adhesion<br>• response to IL-1	1.0E-08<br>1.0E-08<br>1.0E-08	20<br>15<br>9	*Btg2* (3), *Fos* (3), *Adamts* (3), *Egr1* (3), *Klf2* (3)
7	***Jun***^{hi}	• cellular response to IL-1<br>• cellular response to TNF<br>• monocyte chemotaxis	2.5E-04<br>2.5E-04<br>2.5E-04	6<br>7<br>5	Jun (2), *Klf2* (2), *Fos* (2), *Jund* (2), *Btg2* (2)
8	***Nfkbia***^{hi}	• regulation of hemopoiesis<br>• response to IL-1<br>• cytokine biosynthetic process	1.0E-07<br>1.0E-07<br>1.0E-07	20<br>11<br>11	*Nfkbia* (6), *Ccl4* (6), *Ccl2* (5), *Tnf* (4), *Il1b* (2.4)
9	***S100a9***^{hi}	• neutrophil chemotaxis<br>• antigen processing & presentation of exogenous peptide antigen via MHC class II<br>• response to IFN-gamma<br>• astrocyte development	5E-04<br>5E-04<br>5E-04<br>1E-03	6<br>3<br>5<br>3	*S100a9* (2)<br>*S100a8* (2)
10	***Smad7***^{hi}	none			*Smad7* (1.5)
11	***Ctsl***^{hi}	• cellular response to IL-4<br>• antimicrobial humoral immune response	0.01	3<br>0.02	Ribosomal proteins
12	***Hba-a1***^{hi}	none			*Hba-a1* (1.5)<br>*Mir6236* (1.4)<br>*Stat1* (1.3)
13	***Crybb1***^{hi}	none			*Crybb1 (1.4)*<br>*Tuba1a (1.3)*

B

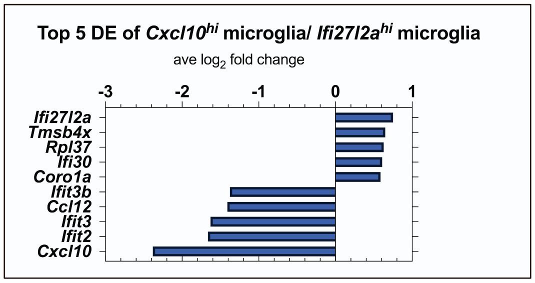

Supplementary Figure 3: Microglia differential expression analysis, Related to Figure 2. A. Gene ontology analysis of the DEGs from each microglia subset compared to all other microglia subsets. Top immune-related gene enrichment pathways distinguishing each microglia subset are shown. The top DEGs of each microglia subset are also shown with the fold-change value shown in parentheses. Data represent an analysis of 102,997 microglia from 11 individual acute TBI and normal mouse brains. **B. Differential expression analysis between *Cxcl10^{hi}* microglia and *Ifi27l2a^{hi}* microglia subsets.** Top DEGs differentially expressed between the two microglia subsets and their ratio of expression in *Cxcl10^{hi}/Ifi27l2a^{hi}* microglia (data are $\log_2$ transformed) are shown.

Supplementary Figure 4

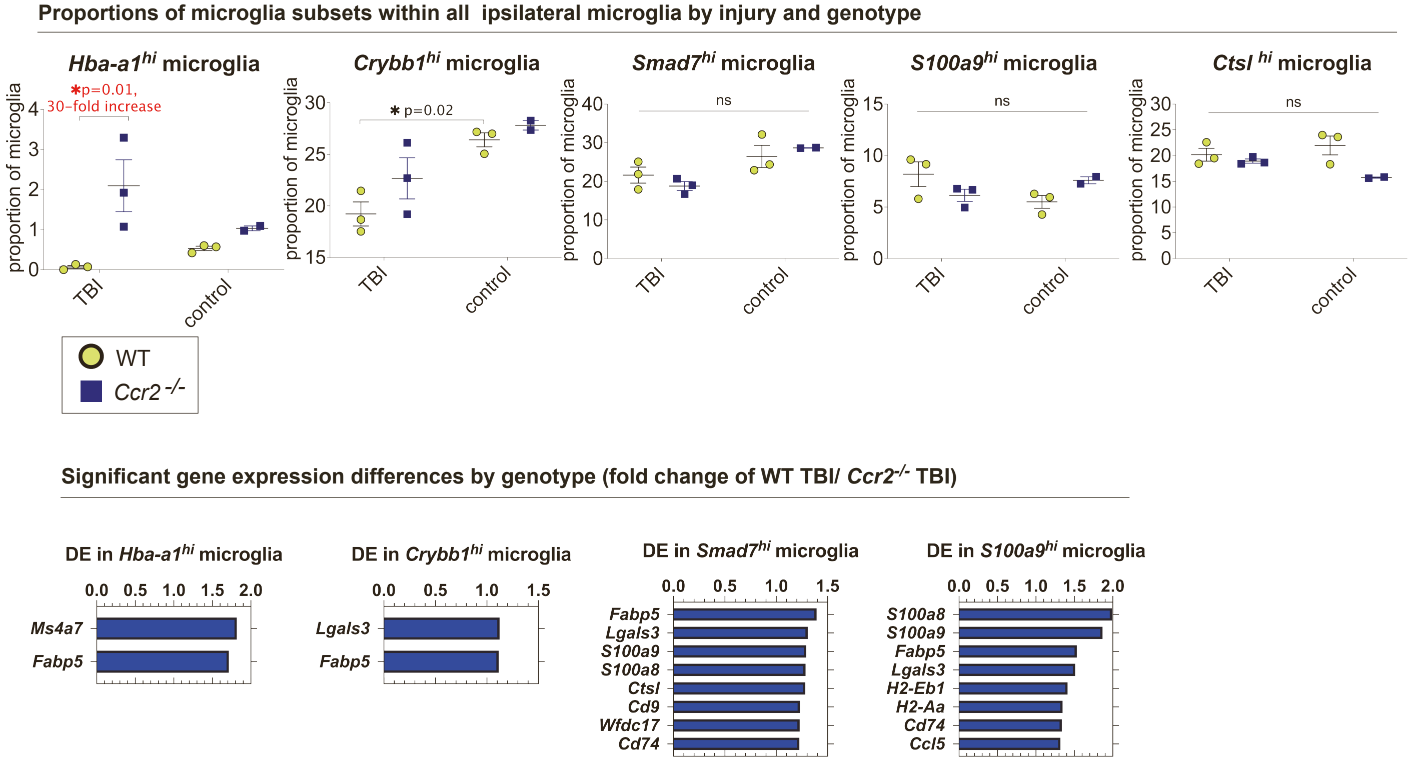

Microglia subset analysis of subsets that were not significantly increased in proportion by acute brain injury, Related to Figure 2. Proportions of each subset as quantified in brain hemispheres by scRNA seq from 11 individual mice (n=2-3/group). Significance was calculated by two way ANOVA and Tukey's multiple comparisons tests. Below: Top signficiant differentially expressed genes by genotype comparing gene expression in WT TBI/*Ccr2*$^{-/-}$ TBI for each microglia subset shown.

Supplementary Figure 5

	Microglia Subcluster	Gene Ontology analysis: Biological processes increased in WT TBI compared to WT normal mice.
1	***Cxcl10**^hi^*	none
2	***Cenpf**^hi^*	• response to IFN-gamma<br>• response to IFN-beta<br>• type I IFN signaling pathway<br>• response to lipopolysaccharide<br>• positive regulation of TNF
3	***Ccl4**^hi^*	• lymphocyte chemotaxis<br>• granulocyte chemotaxis<br>• cellular response to IFN-alpha
4	***Ifi27l2a** ^hi^*	• monocyte chemotaxis<br>• granulocyte chemotaxis<br>• response to IFN-gamma<br>• antigen processing & presentation of peptide antigen
5	***Spp1**^hi^*	• response to IFN-gamma<br>• leukocyte chemotaxis<br>• TNF production<br>• antigen processing & presentation of exogenous peptide via MHC class II<br>• response to IFN-beta
6	***Btg2**^hi^*	• response to IFN-gamma<br>• myeloid leukocyte migration
7	***Jun**^hi^*	• response to IFN-gamma<br>• myeloid leukocyte migration
8	***Nfkbia**^hi^*	• response to IFN-gamma<br>• response to IFN-beta<br>• antigen processing & presentation of peptide antigen<br>• positive regulation of macrophage derived foam cell differentiation
9	***S100a9**^hi^*	• response to IFN-gamma<br>• myeloid leukocyte migration<br>• regulation of viral process
10	***Smad7**^hi^*	• response to IFN-gamma<br>• response to IFN-beta<br>• myeloid leukocyte migration
11	***Ctsl**^hi^*	none
12	***Hba-a1**^hi^*	none
13	***Crybb1**^hi^*	• neutrophil chemotaxis<br>• myeloid leukocyte migration<br>• cellular response to IFN-gamma<br>• endothelial cell proliferation

Gene ontology analysis of significant differentially expressed genes induced by acute TBI, Related to Figure 2. Gene expression in WT TBI microglia were compared to WT normal microglia for each of the 13 identified microglia subsets. Top immune-related gene enrichment pathways elevated in TBI are shown. Data represent an analysis of 102,997 microglia from 11 individual acute TBI and normal mouse brains.

Supplementary Figure 6

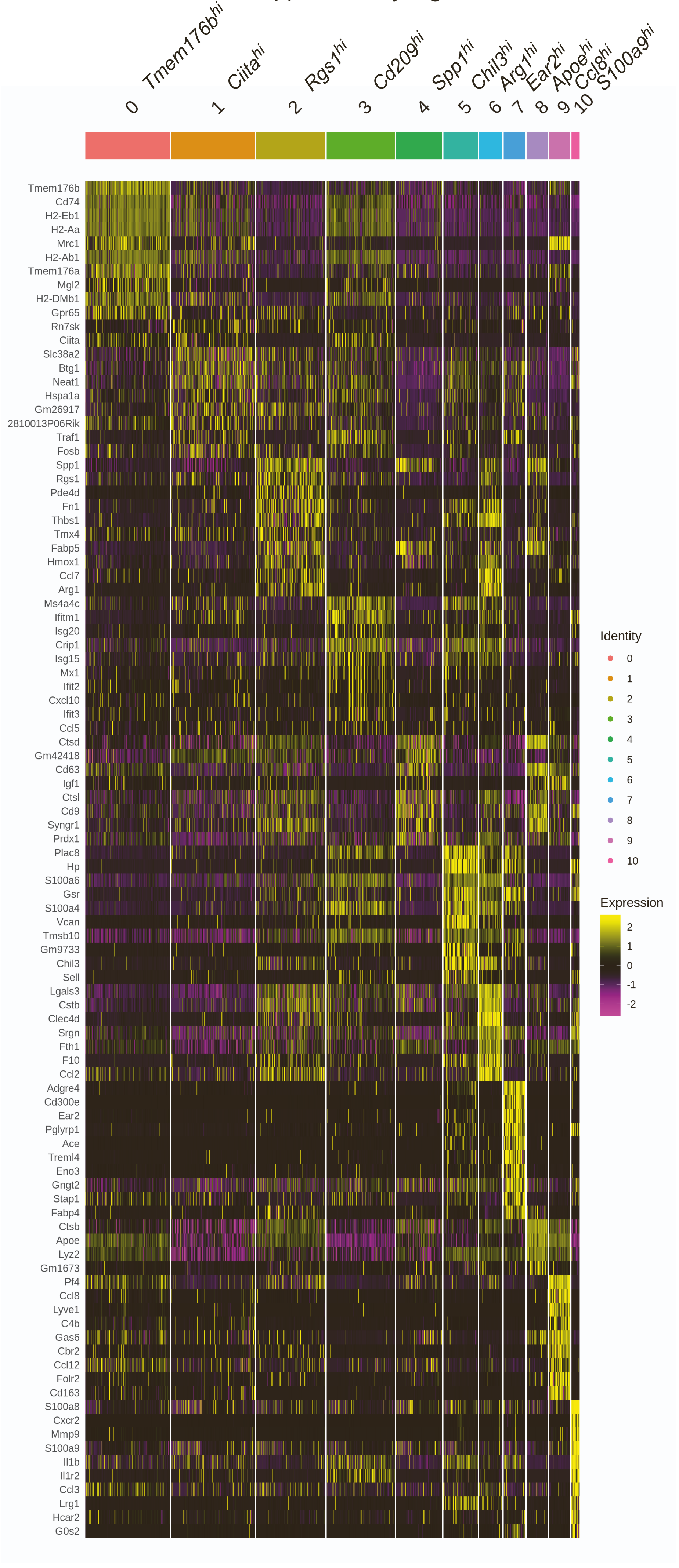

Supplementary Figure 6: Relative expression of significant differentially expressed genes defining each monocyte/macrophage and dendritic cell subcluster found in the acute TBI and normal mouse brains, Related to Figure 4. A heatmap showing relative expression of the top 10 differentially expressed genes between nine monocyte/macrophage subsets and two dendritic cell subsets identified by scRNA seq. Data represent an analysis of 4,076 monocyte/dendritic cells from 11 individual mouse brains (n=3 WT TBI, n=3 *Ccr2*$^{-/-}$ TBI, n=3 WT normal mice, n=2 *Ccr2*$^{-/-}$ normal mice).

Supplementary Figure 7

	Macrophage/ Dendritic Cell Subcluster	Enriched biological processes	q value	#Genes in set	Top DEGs (Fold Change)
**1**	***Chil3 hi***	• response to IFN-beta<br>• myeloid leukocyte migration<br>• granulocyte migration<br>• response to type I interferon	2.5E-05<br>2.5E-05<br>2.5E-05<br>2.5E-05	10<br>16<br>13<br>7	*Plac8* (4), *Chil3* (4), *Hp* (4), *Vcan* (3), *Gsr* (2.4), *Ly6c2* (2), *C3* (2)
**2**	***Arg1hi***	• myeloid leukocyte migration<br>• granulocyte migration<br>• response to IFN-gamma<br>• negative regulation of immune system process<br>• wound healing	1.0E-06<br>1.0E-06<br>1.0E-06<br>1.0E-06<br>1.0E-06	21<br>16<br>15<br>22<br>18	*Arg1* (5), *Lgals3* (3), *Ccl2* (3), *Ccl7* (3), *Ctsb* (3), *Clec4d* (3)
**3**	***Rgs1hi***	• cell chemotaxis<br>• wound healing<br>• myeloid leukocyte migration	1.0E-05<br>1.0E-05<br>1.0E-05	15<br>16<br>14	*Rgs1* (3), *Spp1* (3), *Pde4d* (2), *Arg1* (2)
**4**	***Spp1hi***	• nucleobase-containing small molecule biosynthetic process<br>• ribose phosphate metabolic process	4.0E-06<br>4.0E-06	10<br>12	*Spp1* (3), *Cd63* (2)
**5**	***Tmem176bhi***	• cytoplasmic translation<br>• ribosome biogenesis<br>• antigen processing & presentation of exogenous peptide antigen	1.0E-03<br>1.0E-03<br>1.0E-03	13<br>16<br>6	*Tmem176b* (2.5), *Cd74* (2.5), *H2-Eb1/Aa/ Ab1/DMb1* (2), *Tmem176a* (2), *Mrc1* (2)
**6**	***Ear2hi***	• T cell activation<br>• leukocyte cell-cell adhesion<br>• reactive oxygen species metabolic process	2.0E-06<br>2.0E-06<br>2.0E-06	24<br>20<br>19	*Ear2* (5), *Pglyrp1* (4), *Ace* (4), *Cd300* (4),
**7**	***Apoehi***	• wound healing<br>• protein-lipid complex remodeling<br>• blood coagulation	2.0E-03<br>2.0E-03<br>2.0E-03	9<br>4<br>6	Apoe (4), *Ctsd* (3), *Lyz2* (3), *Ctsb* (3), *Cd63* (2.5)
**8**	***Ccl8hi***	• ribosome biogenesis<br>• cellular response to IL-4<br>• wound healing<br>• response to oxidative stress	2.0E-03<br>2.0E-03<br>4.0E-03<br>4.0E-03	17<br>5<br>10<br>10	*Ccl8* (6), *Pf4* (4), *Mrc1* (3), *Gas6* (3), *Ccl12* (3), *Lyve1* (2.5)
**9**	***S100a9hi***	• cell chemotaxis<br>• myeloid leukocyte migration<br>• granulocyte chemotaxis	1.0E-07<br>1.0E-07<br>1.0E-07	23<br>19<br>16	*S100a9* (21), *S100a8* (17), *Il1b (6)*, *Il1r2 (4)*
**10**	***Cd209hi* dendritic cells**	• response to virus<br>• response to IFN- alpha & beta<br>• response to IFN-gamma<br>• antigen processing & presentation of exogenous peptide antigen via MHCII<br>• T cell activation	2.5E-07<br>2.5E-07<br>2.5E-07<br>2.5E-07<br>2.5E-07	30<br>10-20<br>17<br>6<br>22	*Ifitm1* (3.4), *Isg15* (2), *Ccl5* (2), *Cxcl10* (2), *Ifit2* (2), *Ifit3* (2), *Isg20* (2), *Irf7* (2), *Cd209a* (2)
**11**	***Ciitahi* dendritic cells**	• positive regulation of leukocyte differentiation<br>• regulation of hemopoiesis<br>• positive regulation of cell adhesion<br>• T cell activation	1.0E-04<br>1.0E-04<br>1.0E-04<br>1.0E-04	11<br>15<br>14<br>12	*Hspa1a* (2), *Ciita* (2), *Traf1* (2), *Fos* (2), *Jun* (2)
**12**	***Ccr7hi* dendritic cells**	• negative regulation of immune system process<br>• response to IFN-gamma<br>• regulation of hemopoiesis<br>• positive regulation of cell adhesion<br>• T cell activation	1.0E-08<br>1.0E-08<br>1.0E-08<br>1.0E-08<br>1.0E-08	30<br>19<br>26<br>28<br>28	*Ccl5* (8), *Tbc1d4* (7), *Traf1 (6)* *Ccr7* (5), *Tmem123* (5)

Supplementary Figure 7: Gene ontology analysis of the differentially expressed genes of each monocyte/macrophage and dendritic cell subset, Related to Figure 4. Top immune-related gene enrichment pathways are shown. The top DEGs of each subset are shown with the fold-change value of expression in parentheses. Data represent an analysis of 4,076 monocyte/dendritic cells from 11 individual mouse brains (n=3 WT TBI, n=3 *Ccr2*$^{-/-}$ TBI, n=3 WT normal mice, n=2 *Ccr2*$^{-/-}$ normal mice).

Supplementary Figure 8

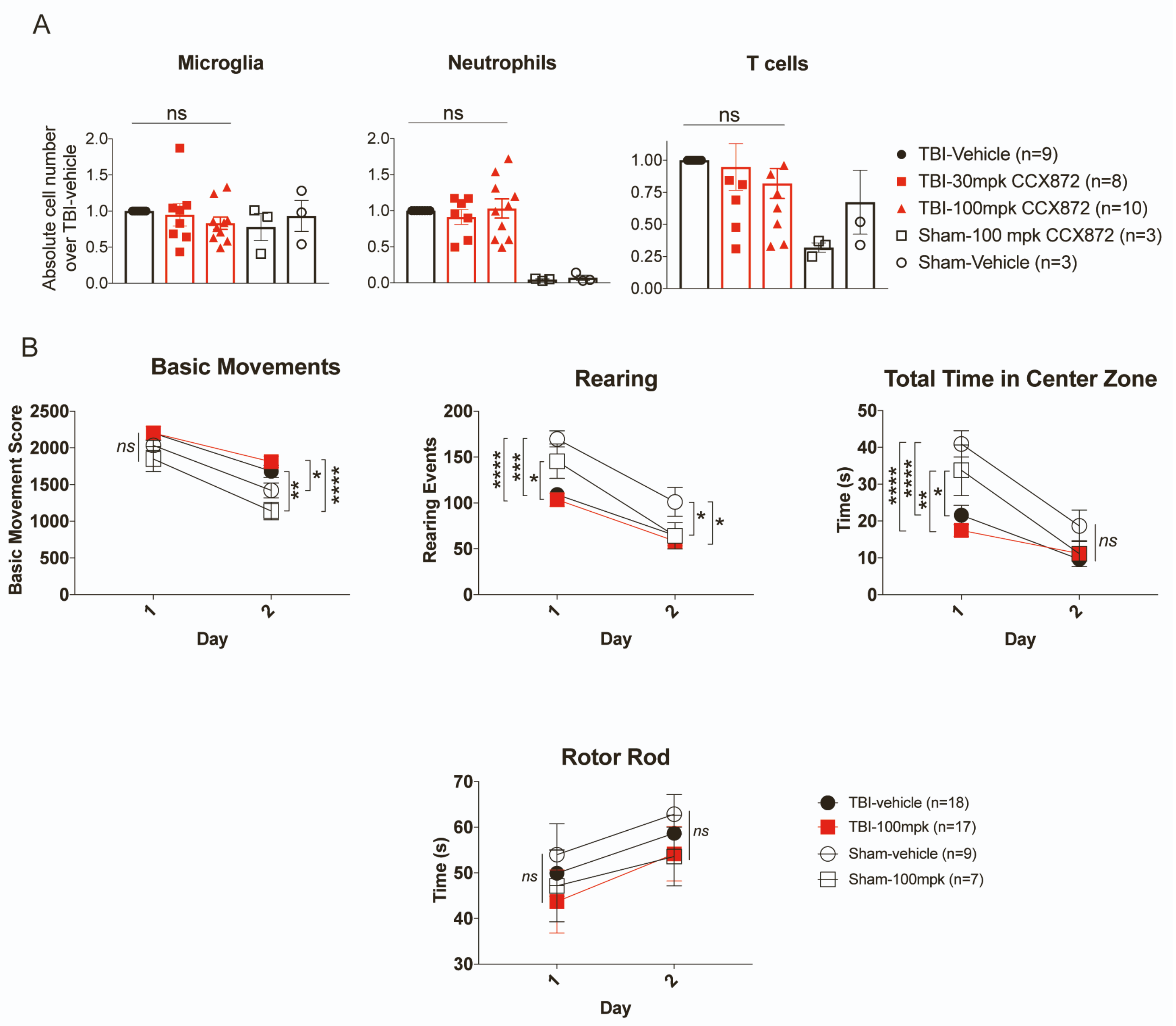

Effects of CCX872 on immune cell numbers in the ipsilateral brain one day post-surgery, and on animal behavior 4 weeks post-surgery, Related to Figure 6. A. hCCR2 knock-in mice treated with a small molecule inhibitor for hCCR2 beginning 2h post-surgery showed no differences in absolute cell numbers of microglia, neutrophils, or CD3+ T cells in the brain one day post-TBI. Flow cytometry analysis and quantification using counting beads determined cell numbers and were plotted as a fold change to the TBI vehicle group (n=8-10/TBI group, n=3/sham group). B. Open field and rotarod testing of hCCR2 knock-in mice treated with a hCCR2 small molecule inhibitor or vehicle. Tests were performed at three weeks post-surgery. Top row: Animals were placed in a novel environment and monitored by a grid of infrared beams for 10 min/day for two days. TBI animals were hyperactive relative to controls as measured by their basic movement score, less exploratory in the vertical direction as assessed by reduced rearing, and more anxious as quantified by their reduced time in the center zone. Bottom row: Motor balance and coordination across all TBI and sham groups were equivalent as measured by similar latencies to stay on the accelerating rotating rod (n=17-18/TBI group, n=7-9/sham group).
